# Signaling Pathways of ESE-16, an Antimitotic and Anticarbonic Anhydrase Estradiol Analog, in Breast Cancer Cells

**DOI:** 10.1371/journal.pone.0053853

**Published:** 2013-01-31

**Authors:** Barend Andre Stander, Fourie Joubert, Chingkuang Tu, Katherine H. Sippel, Robert McKenna, Annie Margaretha Joubert

**Affiliations:** 1 Department of Physiology, University of Pretoria, Pretoria, Gauteng, South Africa; 2 Department of Biochemistry, Bioinformatics and Computational Biology Unit, University of Pretoria, Pretoria, Gauteng, South Africa; 3 Department of Biochemistry and Molecular Biology, College of Medicine, University of Florida, Gainesville, Florida, United States of America; 4 Baylor College of Medicine, Houston, Texas, United States of America; 5 Department of Pharmacology and Therapeutics, University of Florida, Gainesville, Florida, United States of America; Virginia Commonwealth University, United States of America

## Abstract

The aim of this study was to characterize the *in vitro* action of 2-ethyl-3-O-sulphamoyl-estra-1,3,5(10)16-tetraene (ESE-16) on non-tumorigenic MCF-12A, tumorigenic MCF-7 and metastatic MDA-MB-231 breast cancer cells. ESE-16 is able to inhibit the activity of a carbonic anhydrase II and a mimic of carbonic anhydrase IX in the nanomolar range. Gene and protein expression studies using various techniques including gene and antibody microarrays and various flow cytometry assays yielded valuable information about the mechanism of action of ESE-16. The JNK pathway was identified as an important pathway mediating the effects of ESE-16 while the p38 stress-induced pathway is more important in MDA-MB-231 cells exposed to ESE-16. Lysosomal rupture and iron metabolism was identified as important mediators of mitochondrial membrane depolarization. Abrogation of Bcl-2 phosphorylation status as a result of ESE-16 also plays a role in inducing mitochondrial membrane depolarization. The study provides a basis for future research projects to develop the newly synthesized compound into a clinically usable anticancer agent either alone or in combination with other agents.

Keywords: Antimitotic, anticarbonic anhydrase IX, apoptosis, autophagy, cell cycle arrest, Bcl-2, JNK, p38, mitochondrial membrane depolarization, flow cytometry, gene expression and protein microarray, anticancer.

## Introduction

Antimitotic compounds that interfere with the microtubule dynamics in actively dividing cells remain a viable strategy for developing new anticancer agents as evidenced by recent patent applications [1]. Bioavailability and delivery methods of anticancer compounds remain issues that need to be addressed for effective anticancer treatment. 2-Methoxyestradiol (2ME), an antimitotic compound in various phases of clinical trials, suffers from a lack of bioavailability due to the 17-hydroxy group being a target for 17-hydroxysteroid dehydrogenase-mediated metabolism and therefore rapid metabolic breakdown [2]. The 2-methoxyoestradiol-bis-sulphamate analog of 2ME is more resistant to metabolism and its increased bioavaialability is due to its sulphamoyl moieties [3]. Improved oral bioavailability is argued to be as a result of the potential of aryl sulphamoyl containing compounds to reversibly bind to carbonic anhydrase II present in red blood cells and in turn circumvent first pass liver metabolism [4]. ENMD-1198, another analog of 2ME is undergoing clinical trials and the D-ring modification appears to improve bioavailability when compared to 2ME [5], [6], [7], [8], [9], [10].

2-Ethyl-3-O-sulphamoyl-estra-1,3,5(10)16-tetraene (ESE-16) was previously identified as an antimitotic compound and the 16-dehydration found in ESE-16 corresponds with ENMD-1198 [9], [11]. ESE-16 was synthesized due to its potential antimitotic as well as carbonic anhydrase IX (CAIX) inhibitory activity. The metabolic environment in solid tumors has several characteristics including acidosis [12]. CAIX, an extracellular carbonic anhydrase isoenzyme, is over expressed in a variety of tumors and contributes to the acidification of the extracellular microenvironment by catalyzing the conversion of carbon dioxide and water to carbonic acid [4], [13]. Acidic extracellular pH in turn contributes the breakdown of the basement membrane as well as the induction of the expression of proteinases which facilitate invasion and metastasis [14], [15]. Carbonic anhydrase II is an ubiquitously expressed intracellular carbonic anhydrase [16]. Selective inhibition of CAIX provides a valuable strategy for curtailing the development of metastatic processes associated with acidic microenvironmental conditions in tumors.

Since the exact mechanism of action of ESE-16 remains to be elucidated, the purpose of this study was to investigate the *in vitro* influence of ESE-16 in non-tumorigenic MCF-12A, tumorigenic MCF-7 and metastatic MDA-MB-231 breast cancer cells. Data obtained from the present study demonstrate the influence of ESE-16 on carbonic anhydrase II and IX-mimic kinetics, gene and protein expression, cell morphology, the generation of reactive oxygen species, lysosomal stability, apoptosis induction, mitochondrial membrane potential, Bcl-2 phosphorylation and caspase activity. We demonstrate that ESE-16 inhibits CAII in the nanomolar range and is more selective towards a mimic of carbonic anhydrase IX. The data from this study yielded valuable information about the mechanism of action of ESE-16 on various breast cell lines. It is well known that mitotic arrest due to antimitotic treatment leads to the activation of stress-activated protein kinases (SAPKs) p38 and JNK [17]. The JNK pathway appears to be more important than the p38 pathway in MCF-7 cells, while the p38 pathway seems to be more important in MDA-MB-231 and MCF-12A cells in mediating the pro-apoptotic events induced by ESE-16. Lysosomal rupture and iron metabolism were identified as important mediators of cell death in ESE-16-treated cells. Several testable hypotheses regarding the mechanism of action of ESE-16 were generated from the data, including identifying the unfolded protein response as a potentially causal factor in inducing cell death due to ESE-16 exposure. Altogether, the study provides a basis for future research projects to develop the newly synthesized compound into a clinically usable anticancer agent.

## Materials and Methods

### Materials

The tumorigenic estrogen receptor positive MCF-7, metatstatic estrogen receptor negative MDA-MB-231 and the non-tumorigenic estrogen receptor negative MCF-12A cell lines were obtained from the American Type Culture Collection (ATCC, Manassas, Virginia, USA). Heat-inactivated fetal calf serum (FCS), sterile cell culture flasks and plates were obtained through Sterilab Services (Kempton Park, Johannesburg, South Africa). Dulbecco’s minimum essential medium Eagle (D-MEM), penicillin, streptomycin, and fungizone were purchased from Highveld Biological (Pty) Ltd. (Sandringham, South Africa). The Nanodrop, an Axon Genepix 4000B Scanner and Agilent’s Sure- Hyb chambers at the African Centre of Gene Technology (ACGT) Microarray Facility (University of Pretoria, Pretoria, South Africa) were purchased from Inqaba Biotechnical Industries (Pty) Ltd. (Pretoria, SA), Molecular Devices Corporation, (Sunnyvale, CA, USA) and Agilent Technologies (Pty) Ltd. (Palo Alto, CA, USA), respectively. The fluorescence activated cell sorting (FACS) FC500 System flow cytometer equipped with an air-cooled argon laser excited at 488 nm was purchased from Beckman Coulter South Africa (Pty) Ltd. (Pretoria, South Africa). Agilent’s 44k 60-mer human oligo slides, Low RNA Input Fluorescent Linear Amplification Kit, 2× GEx Hybridization Buffer HI-RPM, Gene Expression (GE) Wash Buffer 1 and 2 and the Stabilization and Drying Solution were purchased from Agilent Technologies (Pty) Ltd. (Palo Alto, CA, USA). All other chemicals were of analytical grade and were purchased from Sigma Chemical Co. (St. Louis, MO, USA). Antibody Microarray 500 slides, Protein Extraction and Labeling Kit and the Ab Microarray Express Buffer Kit were obtained from Clontech Laboratories, Inc. (Mountain View, CA, USA). A bicinchoninic acid (BCA) Protein Assay Reagent Kit was obtained from Pierce Biotechnology (Rockford, Illinois, USA). Zeba Spin Desalting Columns, 7K MWCO were purchased from Thermo Fisher Scientific (Waltham, Massachusetts, USA). Cy5 mono-Reactive Dye and Cy3 mono-Reactive Dye Packs were purchased from GE Healthcare (Johannesburg, South Africa). The Mitocapture™ apoptosis detection kit and a rabbit antibody for anti-active caspase 7 were obtained from BioVision Inc. (Mountain View, California, USA). A rabbit antibody for anti-active caspase 3 was supplied by IMGENEX (San Diego, California, USA). An anti-rabbit antibody conjugated to Dylight™ 488 was purchased from Rockland Inc (Gilbertsville, Pennsylvania, USA). The FlowCellect Bcl-2 Activation Dual Detection Kit was purchased from Millipore Corporation (Billerica, Massachusetts, USA). An RNeasy kit and RNase-free DNase were purchased from Qiagen (Valencia, CA, USA). All other chemicals were of analytical grade and were purchased from Sigma Chemical Co. (St. Louis, MO, USA).

### Materials and Methods - Cell Culture and Maintenance

MDA-MB-231 (estrogen receptor negative) tumorigenic and metastatic breast cancer cells and MCF-7 (estrogen receptor positive) breast cancer cells were cultured in DMEM and supplemented with 10% heat-inactivated FCS (56°C, 30 min), 100 U/ml penicillin G, 100 µg/ml streptomycin and fungizone (250 µg/l). MCF-12A maintenance medium consisted of a 1∶1 mixture of DMEM and Ham’s-F12 medium, 20 ng/ml epidermal growth factor, 100 ng/ml cholera toxin, 10 µg/ml insulin and 500 ng/ml hydrocortisone, supplemented with 10% heat-inactivated FCS (56°C, 30 min), 100 U/ml penicillin G, 100 µg/ml streptomycin and fungizone (250 µg/l). ESE-16 was dissolved in dimethyl sulphoxide (DMSO). The final concentration of DMSO did not exceed 0.05% in cell culture. Experiments were conducted in 96-well, 6 well plates or 25 cm^2^ cell culture flasks. Exponentially growing cells were seeded at 5000 and 250 000 cells per well for 96-well, 6 well plates respectively. Cells were seeded at 750 000 cells per 25 cm^2^ cell culture flask. After a 24 h incubation period at 37°C to allow for cell adherence, cells were exposed to the compounds including the vehicle-control and incubated for 12 h, 24 h or 48 h at 37°C.

### Carbonic Anhydrase II and IX Mimic Kinetics

The oxygen-18 (^18^O) method for monitoring reaction velocity was employed to determine the inhibition constants (K_i_) of ESE-16 on CAII and a CAIX mimic [18]. Membrane inlet mass spectrometry measures the rate of depletion of ^18^O from species of CO_2_. For carbonic anhydrase, the reaction velocity (R_1_/[E]) is for the rate of exchange between ^18^O-labeled carbonate to carbon dioxide and zinc-bound ^18^O-labeled hydroxide. The weighted average of the tight-binding inhibition constant K provides the K_i_ and is calculated using the Henderson method for tight-binding inhibitors [19].
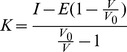



*V*_0_ is the reaction velocity (R_1_/[E]) observed in the absence of an inhibitor.

*V* is the reaction velocity (R_1_/[E]) observed at the concentration of an inhibitor *I*.

*E* is the active enzyme concentration (7.3 nM).

Experiments were performed at 25°C in 0.1 M HEPES, pH7.4, 10 mM total carbonate concentration and inhibitor concentrations ranged up to 8 µM.

The pH of the extracellular growth medium of confluent MDA-MB-231 cells exposed to Desferoxamine (DFO), ESE-16 and a combination of DFO and ESE-16 for 24 h was measured in order to evaluate the ability of ESE-16 to prevent extracellular acidification by inhibiting CAIX activity [20]. An Orion 3 Star Benchtop pH meter from Thermo Fisher Scientific (Waltham, Massachusetts, USA) was used for the pH measurement of extracellular growth medium.

### Global Gene and Protein Expression Analysis: Complimentary RNA Microarray and Protein Microarray

Agilent’s Human 1A Oligo 60-mer Microarray (V2) 44 k slides with more than 41 000 60-mer oligonucleotide human genes and transcripts were employed to study global expression changes on the MCF-12A, MCF-7 and MDA-MB-231 cell lines induced after 24 h treatment of ESE-16. A dye-swop methodology with three biological replicates was employed in order to remove the effects of dye-bias on statistical analyses and make the genomic information statistically relevant. Detailed RNA microarray methods are explained in Supplementary Information S1.

Antibody microarrays can help provide a means to measure global protein expression levels in whole cell extracts [21]. The technique was employed to yield valuable mechanistic information with regards to signaling mechanisms being activated or abrogated in treated samples when compared to control samples [22]. The technique involves the spotting of a slide with a library of antibodies relevant to a particular functional pathway being studied. Proteins in whole protein extracts are labeled with fluorescent dyes and hybridized onto the antibody protein microarray. The relative fluorescence of each spot provides an indication of the relative abundance of the protein in control and treated samples. Detailed methods are explained in Supplementary Information S1.

### Morphological Examination of Intracellular Ultrastructure

Acridine orange (AO) (green) and Hoechst 33345 (blue) fluorescent dyes were used to qualitatively monitor the intracellular distribution of AO and nuclear morphology respectively. AO emits green light when present in the cytosol provides an indication of lysosomal activity [23], [24]. Hoechst 33342 is a fluorescent dye that can penetrate intact cell membranes of viable cells and cells undergoing apoptosis and stain the nucleus. Time-dependent studies were carried out at intervals of 24 h and 48 h. The cells were examined with a Zeiss inverted Axiovert CFL40 microscope and Zeiss Axiovert MRm monochrome camera under Zeiss Filter 2 for Hoechst 33342 (blue) stained celssand Zeiss Filter 9 for acridine orange-stained cells (green) (Carl Zeiss (Pty) Ltd., Johannesburg, South Africa). In order to prevent fluorescent dye quenching, all procedures were performed in a dark room.

### Flow Cytometry

Flow cytometry was employed to analyze redox status, lysosomal stability, apoptosis, mitochondrial membrane potential, the phosphorylation status of Bcl-2 at Ser 70, and caspase 3- and caspase 7 expression. 2,7-dichlorofluorescein (DCF) was used to measure the redox status of cells. Redox status was monitored with DCFDA (2,7-dichlorofluorescein diacetate). Cell permeable DCFDA is intracellularly cleaved to DCF which in turn is oxidized to a green fluorescent molecule in the presence of redox-active transition metals and H_2_O_2_ and thus an indirect indicator of intracellular redox status [25]. Acridine orange was used to measure the functional state of lysosomes. AO is lysosomotropic and emits red light at high concentrations (when present in functional lysosomes) and green light when present in the cytosol [23], [24]. For apoptosis analysis, cells were stained with FITC-conjugated Annexin V to measure the translocation of the membrane phosphatidylserine (PS). PS translocation is associated with apoptotic processes. Time-dependent studies were carried out at intervals of 6 h, 12 h, 18 h, 24 h and 48 h.

Mitochondrial membrane potential was monitored using Mitocapture™. Mitocapture™ is a cationic dye that accumulates and aggregates in the mitochondria of healthy cells, providing a bright red fluorescence [26]. MitoCapture™ cannot aggregate in the mitochondria due to a reduction in mitochondrial membrane potential and thus remains in the cytoplasm in its monomer form, generating a green fluorescence [26]. Time-dependent studies were conducted at intervals of 6 h, 12 h, 18 h, 24 h and 48 h. In order to gain further mechanistic insight into the effects that stress activated protein kinases have on mitochondrial membrane potential, cells were exposed to inhibitors of the selective JNK kinase inhibitor, SP600125 and the selective p38 MAP kinase inhibitor, SB239063. The effect that the ferric iron chelator Desferoxamine (DFO, 100 µM [20]) and the ferrous iron 1,10-Phenanthroline (Phen, 25 µM [27]), as well as the antioxidant *N*-acetyl cysteine (NAC) had on mitochondrial membrane potential was assessed on MDA-MB-231 cells. Active caspase 3 and 7 are effector caspases that cleave a number of substrates resulting in morphological and biochemical features of apoptosis [28]. Primary antibodies against active caspase 3 and 7 were used to study protein expression changes in the MCF-7, MDA-MB-231 and MCF-12A cell lines after 24 h treatment of ESE-16.

Flow cytometry was employed to study the phosphorylation status of Bcl-2 at serine 70 as well as the overall Bcl-2 protein expression in the MCF-12A, MCF-7 and MDA-MB-231 cell lines 24 h treatment of selected compounds. JNK and p38 MAP kinase inhibitor s SP600125 and SB239063 were used to gain further mechanistic insight into the effects that stress activated protein kinases have Bcl-2 phosphorylation. Detailed methods are explained in Supplementary Information S1.

### Statistics

Data was obtained from 3 independent experiments. Obtained data was statistically analyzed for significance using a two-tailed Student’s *t*-test. Means are presented in bar charts, with T-bars referring to standard deviations. Measurement of fluorescence via flow cytometry was expressed as a ratio of the value measured for vehicle-treated exposed cells (relative fluorescence).

## Results

### In vitro Carbonic Anhydrase Inhibition

**Figure 1:**
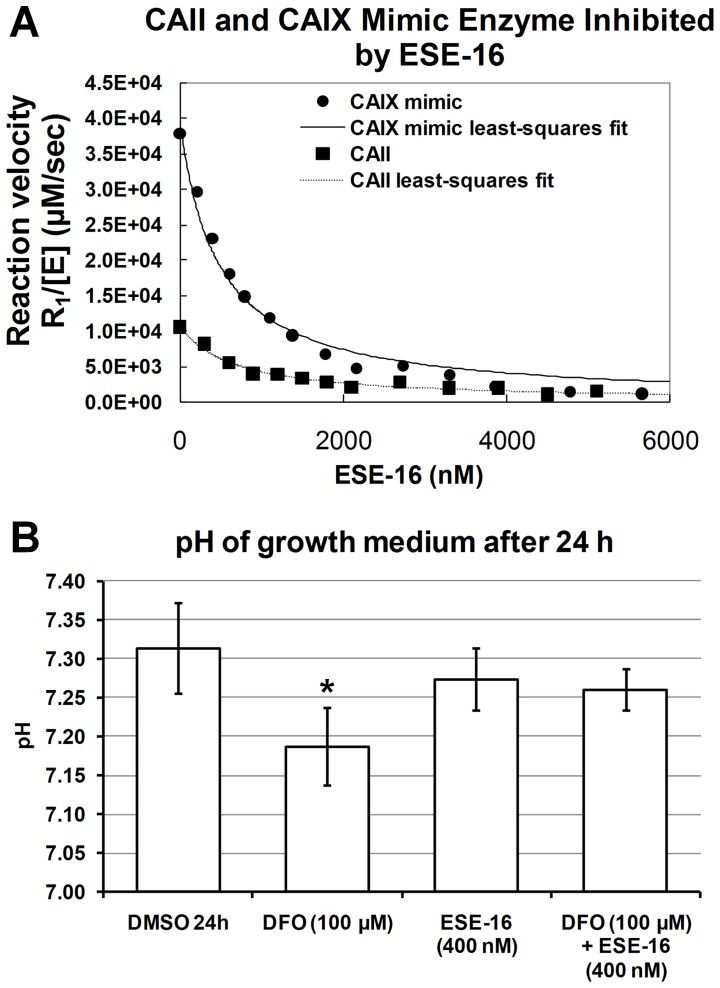
Kinetics data of ESE-16 and the effects of ESE-16 on extracellular acidity in MDA-MB-231 cells after 24 h exposure. A) Variation with concentration of ESE-16 of the reaction velocity (R_1_/[E]) of wild-type CAII and a mimic of CAIX as determined by the catalysis of ^18^O exchange. Wild-type CAII K_i_ = 569±61 nM and CAIX mimic K_i_ = 453±43 nM, calculated using the Henderson method for tight-binding inhibitors. B) Changes in extracellular pH of confluent MDA-MB-231 cells treated with the CAIX inducer, DFO, and ESE-16 and a combination of DFO and ESE-16. ESE-16 inhibited DFO-induced reduction in extracellular pH.

ESE-16 was synthesized as a CAIX inhibitor that can selectively inhibit the development of metastatic processes due extracellular acidification by CAIX. Carbonic anhydrase inhibition of the physiologically dominant CAII and a mimic of the tumor-associated CAIX were assessed using gas inlet mass spectroscopy. The accuracy of CAIX mimic as a model of wild-type CA provides a useful tool to test isozyme-specific CA IX inhibitors [29], [30]. Kinetics data indicated that ESE-16 inhibited the CAIX mimic (K_i_ = 453±43 nM) at a lower concentration when compared to the wild-type CAII (K_i_ = 569±61 nM) (Figure 1A).

Li *et al.* (2009) have demonstrated that CAIX protein expression is induced in response to the iron chelator, Desferoxamine (DFO), in MDA-MB-231 cells and that this contributes to extracellular acidification [20]. In order to test whether ESE-16 can inhibit extracellular acidification due to CAIX over expression, confluent MDA-MB-231 cells were exposed to ESE-16 with and without DFO for 24 h. After 24 h exposure, a statistically significant decrease in pH of the growth medium in DFO-treated cells was observed while confluent MDA-MB-231 cells treated with DFO in conjunction with ESE-16 (400 nM) did not (Figure 1B). The data suggest that ESE-16 is capable of abrogating extracellular acidification due to CAIX expression.

### Global Gene and Protein Expression Analysis

**Figure 2:**
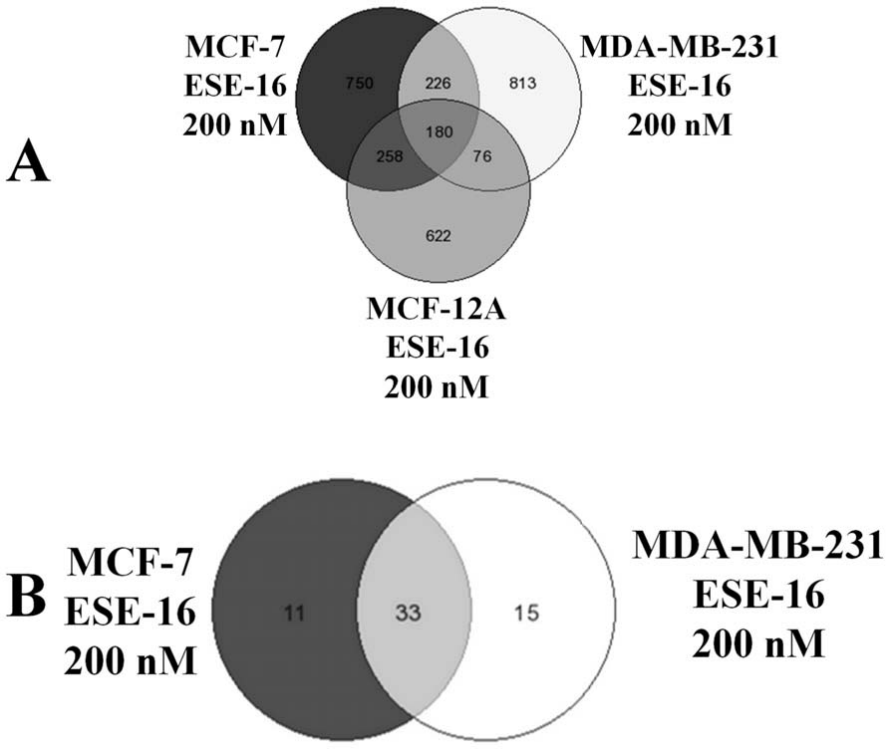
GeneVenn diagram showing of statistically significant differentially expressed genes and proteins in MCF-7, MDA-MB-231 and MCF-12A cells after 24 h exposure. Common genes (A) affected in MCF-7, MDA-MB-231 and MCF-12A cells, and common proteins (B) affected in MCF-7 and MDA-MB-231 cells exposed to ESE-16 (200 nM) for 24 h.

Agilent’s Human 1A Oligonucleotide Microarray was employed to collect information about gene expression changes that associated with ESE-16-exposed cells. MCF-7, MDA-MB-231 and MCF-12A cells were exposed to ESE-16 (200 nM) for 24 h cells. For ESE-16-treated cells, 1414, 1295 and 1136 genes were considered to be statistically significantly differentially expressed in MCF-7, MDA-MB-231 and MCF-12A cells respectively with 180 genes differentially expressed all the ESE-16-exposed cells (Figure 2). Genes that were considered statistically significantly differentially expressed (adjusted *P-*value <0.05) in treated cells were mapped to genes associated with cell growth, apoptosis, autophagy, cell cycle and DNA repair, oxidative stress, phosphatases, kinases, epigenetics-related and chromatin modifications, structural, regulation of transcription and translation, Ras-related, extracellular matrix and heat-shock protein related (Table 1 and Supplementary Information S2).

**Table 1:** Differentially expressed genes (adjusted *P-*value <0.05) mapped to functional cellular pathways in MCF-7, MDA-MB-231 and MCF12A cells exposed ESE-16 (200 nM) for 24 h.

Refseq RNA	Gene name	Description	Log M (Diff expressed)		
			ESE-16	ESE-16	ESE-16
			MCF-7	MDA-MB-231	MCF-12A
			200 nM	200 nM	200 nM
**Cell growth and cell death**					
NM_014417	BBC3/PUMA	BCL2 binding component 3	1.66	1.7	1.81
NM_001731	BTG1	B-cell translocation gene 1 anti-proliferative	1.32	0.78	0.45
NM_004417	DUSP1	Dual specificity phosphatase 1	1.97	2.05	1.33
NM_033285	TP53INP1	Tumor protein p53 inducible nuclear protein 1	1.18	0.74	0.99
NM_006533	MIA	Melanoma inhibitory activity	0.43	0.66	0.62
NM_006879	MDM2	Mdm2 transformed 3T3 cell double minute 2	1.23	1.85	1.13
NM_138764	BAX	BCL2-associated X protein transcript variant epsilon			0.49
NM_001343	DAB2	Disabled homolog 2 mitogen-responsive phosphoprotein	0.78		0.59
NM_001924	GADD45A	Growth arrest and DNA-damage-inducible alpha	0.41	0.66	
**Cell cycle and DNA repair**					
NM_001007793	BUB3	Budding uninhibited by benzimidazoles 3 homolog	−1.32	−0.78	−0.45
NM_001786	CDC2	Cell division cycle 2 G1 to S and G2 to M	−0.91	−1.24	−0.84
NM_030928	CDT1	Chromatin licensing and DNA replication factor 1	−0.52	−0.96	−0.56
NM_006622	PLK2	Polo-like kinase 2	0.96	1.23	0.99
NM_020675	SPBC25	Spindle pole body component 25 homolog	−0.75	−0.69	−0.55
NM_006879	MDM2	Mdm2 transformed 3T3 cell double minute	1.23	1.85	1.13
NM_079836	TUBA2	Tubulin, alpha 2	−1.11	−0.56	−1.11
NM_006088	TUBB2C	Tubulin beta 2C	−1.32	−0.8	−1.09
NM_013282	UHRF1	Ubiquitin-like containing PHD and RING finger domains 1	−0.99	−1.59	−1.06
NM_002358	MAD2L1	MAD2 mitotic arrest deficient-like 1		−0.59	−0.48
NM_006341	MAD2L2	MAD2 mitotic arrest deficient-like 2	−0.38		−0.41
NM_002577	PAK2	p21 -activated kinase 2	−0.51	−0.78	
NM_203401	STMN1	Stathmin 1/oncoprotein 18	−0.33	−0.8	
**Oxidative stress related**					
NM_002133	HMOX1	Heme oxygenase 1	0.77	1.15	0.95
NM_002970	SAT1/SSAT	Spermidine/spermine N1-acetyltransferase 1	0.8	0.81	0.68
NM_175839	SMOX	Spermine oxidase		0.91	
NM_001024465	SOD2	Superoxide dismutase 2		1.1	
NM_000625	NOS2A	Nitric oxide synthase 2A		0.75	0.36
NM_002032	FTH1	Ferritin, heavy polypeptide 1	0.51	0.65	
**Regulation of Transcription**					
NM_002128	HMGB1	High-mobility group box 1	−0.67	−0.7	−0.75
NM_000600	IL6	Interleukin 6	1.68	3.66	1.93
X66087	MYBL1	V-Myb myeloblastosis viral oncogene homolog (avian)-like 1	−0.7	−1.03	−0.94
NM_172164	NASP	Nuclear autoantigenic sperm protein	−0.46	−0.64	−0.44
NM_021005	NR2F2	Nuclear receptor subfamily 2 group F member 2	−0.91	−1.33	−0.77
NM_006509	RELB	v-Rel reticuloendotheliosis viral oncogene homolog B	0.77	1.06	0.74
NM_182898	CREB5	Camp responsive element binding protein 5 transcript variant 1	0.47	0.71	0.52

Antibody microarrays were used to determine protein expression changes in response to ESE-16 treatment after 24 h exposure [21]. After analysis, 44 proteins were considered differentially expressed in MCF-7 cells and 48 proteins were considered differentially expressed in MDA-MB-231 cells after 24 h exposure to 200 nM of ESE-16 (Figure 2). Of these proteins, 33 were differentially expressed in both cell lines indicating a shared proteomic response to ESE-16 exposure in both MCF-7 and MDA-MB-231 cells (Figure 2, Table 2).

**Table 2:** Expression of proteins deemed statistically significantly (standard deviation of less than 5% of *Average Log M* or has an *Average Log M* of greater than ±0.2) differentially expressed proteins as determined by antibody array analyses in MCF-7 and MDA-MB-231 cells exposed to ESE-16 (200 nM) for 24 h.

RefSeq RNA	Gene ID	Protein ID	Description	MCF-7		MDA-MB-231	
				Average Log_2_	STDEV	Average Log_2_	STDEV
**Cell death**							
BAX	581	Q07814	BCL2-associated X protein			**0.23**	0.067
EPB49	2039	Q08495	Erythrocyte membrane protein band 4.9 (dematin)	**0.77**	<0.001	**0.63**	0.115
CASP4	837	P49662	Caspase 4, apoptosis-related cysteine protease			**0.32**	0.001
CASP7	840	P55210	Caspase 7, apoptosis-related cysteine protease			**0.23**	0.06
STAT3	6774	P40763	Signal transducer and activator of transcription 3	**0.23**	0.062	**0.46**	0.071
DAB2	1601	P98082	Disabled homolog 2, mitogen-responsive phosphoprotein	**0.47**	0.022		
**Cell cycle**							
CCNB1	891	P14635	Cyclin B1			**0.15**	0.001
BUB3	9184	O43684	BUB3 budding uninhibited by benzimidazoles 3			**0.34**	0.144
**Protein folding**							
HSP60	3329	P10809	Heat shock 60 kDa protein 1 (chaperonin)	**0.2**	0.05		
HDJ-2	3301	P31689	DnaJ (Hsp40) homolog, subfamily A, member 1	**0.31**	0.05	−**0.24**	0.04
HSPA1A	3303	P08107	Heat shock 70 kda protein 1A			**0.32**	0.053
**Ras-related**							
CSK	1445	P41240	c-Src tyrosine kinase	**0.27**	0.004	**0.19**	0.017
ARHGEF7	8874	Q14155	Rho guanine nucleotide exchange factor (GEF) 7	−**0.29**	0.035	−**0.21**	0.073
ARHGDIB	397	P52566	Rho GDP dissociation inhibitor (GDI) beta	**0.24**	0.008		
RASA2	5922	Q15283	RAS p21 protein activator 2			**0.27**	0.062
RAC1	5879	P15154	Ras-related C3 botulinum toxin substrate 1			−**0.44**	0.062
Ras-GAP	5921	P20936	RAS p21 protein activator (GTPase activating protein) 1			**0.2**	0.12
**Transcription and translation**							
TCERG1	10915	O14776	Transcription elongation regulator 1 (CA150)	**0.28**	0.013	**0.29**	0.019
NASP	4678	P49321	Nuclear autoantigenic sperm protein (histone-binding)	−**0.27**	0.013	−**0.58**	0.025
RPS6KB1	6198	P23443	Ribosomal protein S6 kinase, 70 kda, polypeptide 1			−**0.12**	0.011
NCOR2	9612	Q9Y618	Nuclear receptor co-repressor 2	−**1.11**	0.127		

Several cell death-associated, cell stress-responsive and p53-responsive genes were differentially expressed including disabled homolog 2 mitogen-responsive phosphoprotein (DAB2), DNA-damage-inducible alpha (GADD45A), p53 up regulated modulator of apoptosis (PUMA/BBC3), tumor protein p53 inducible nuclear protein 1 (TP53INP1), the murine double minute 2 oncogene (mdm2) and diacylglycerol kinase alpha (DGKA), heme oxygenase 1 (HMOX1), dual specificity phosphatase 1 (DUSP1/MK*P-*1), DNA-damage-inducible transcript 3 (DDIT/CHOP/GADD153) and spermidine/spermine acetyltransferase (SSAT) (Table 1 and Supplementary Information S2). DAB2 is a tumor suppressor and interacts with DAB2 interacting protein (DAB2IP, DIP1/2, AIP1) in a manner that enhances its tumor suppressing activity [31], [32]. Both mRNA and protein expression of disabled homolog 2 mitogen-responsive phosphoprotein (DAB2) were up regulated in ESE-16-treated MCF-7 cells but not in MDA-MB-231 cells. Both BBC3 and GADD45A are stress-inducible proteins whose gene expression is transcriptionally activated by active tumor suppressor protein p53 [33], [34], [35]. TP53INP1, mdm2 and DGKA are transcriptionally activated by p53 [36].

DNA-damage-inducible transcript 3 (DDIT/CHOP/GADD153) gene expression was up regulated in all the tested cell lines exposed to ESE-16 (Table 1). DDIT3 is a cellular stress-inducible proteins and is a key player in response to endoplasmic reticulum stress associated with unfolded proteins [37], [38]. Several heat shock proteins and chaperonins genes were differentially expressed in ESE-16-treated cells. Heat shock protein 90 alpha (HSP90α) gene expression was down regulated in MCF-7, MDA-MB-231 and MCF-12A cells while heat shock protein 90 beta (HSP90β) gene expression was down regulated in MCF-7 and MCF-12A cells exposed to ESE-16 (Table 1, Table 2 and Supplementary Information S2). Several other heat shock proteins from the HSP70 family were also differentially expressed including heat shock 70 kDa protein 2 (HSPA2, down regulated in MCF-7 and MDA-MB-231 cells), -5 (HSPA5, down regulated in MDA-MB-231 cells) and -6 (HSPA6, up regulated in MCF-7 cells) (Table 1, Table 2 and Supplementary Information S2). Several chaperonins genes from the DnaJ (HSP40) subfamilies B and C were differentially expressed. Protein expression studies revealed that heat shock 60 kDa protein 1 (down regulated in MCF-7 cells), DnaJ (Hsp40) homolog, subfamily A, member 1 (down regulated in MCF-7 and up regulated in MDA-MB-231 cells) and heat shock 70 kda protein 1A (up regulated in MDA-MB-231 cells) were differentially expressed in response to ESE-16 exposure (Table 1, Table 2 and Supplementary Information S2). The Hsp70-Hsp90 Organizing stress-induced-phosphoprotein (HOP/STIP1) gene was down regulated in MCF-7 and MCF-12A cells (Table 1 and Supplementary Information S2). Protein array studies suggest that caspase 4 was also up regulated in MDA-MB-231 cells exposed to ESE-16 (Table 2). Caspase 4 is the human homolog of murine caspase 12 and is localized to the ER membrane and is specifically activated by and required for ER stress-induced apoptosis [39]. Together, these results suggest that the unfolded protein response (UPR) may play a role in inducing cell death in ESE-16-treated cells [37], [38]. However, further studies are needed to confirm this.

The mitotic checkpoint complex (MCC) is activated in response to abnormal mitotic spindle formation during mitosis [40], [41], [42], [43]. When active, the MCC inhibits the activity of the anaphase promoting complex (APC/C-cdc20) complex which in turn prevents the onset of anaphase due to CDK1/cyclin B activity [40], [41], [42], [43]. Decreased expression of cyclin B and increased protein levels of cyclin B was observed in MDA-MB-231 cells while CDK1 gene expression was down regulated in all cell lines exposed to ESE-16 (Table 1, Table 2 and Supplementary Information S2). Previous studies on 2ME, another antimitotic estradiol analog, have revealed similar results whereby up regulated of CDK1 activity is accompanied with increased cyclin B protein expression and decreased cyclin B and CDK1 gene expression [44], [45], [46], [47].

Several genes involved in microtubule dynamics and the mitotic checkpoint complex (MCC) were differentially expressed ESE-16-treated cells. Genes that were down regulated in all cell lines in response to ESE-16 exposure include include budding uninhibited by benzimidazoles 3 (BUB3) and spindle pole body component 25 (SPC25) (Table 1 and Supplementary Information S2). Polo-kinase 2 (PLK2) gene expression was up regulated in all ESE-16 exposed cell lines (Table 1 and Supplementary Information S2). Stathmin 1/oncoprotein 18 (STMN1) gene expression was down regulated in MCF-7 and MDA-MB-231 cells (Table 1 and Supplementary Information S2). MAD2 mitotic arrest deficient-like 1 (MAD2L1) was down regulated in MDA-MB-231 and MCF-12A cells and MAD2 mitotic arrest deficient-like 2 was down regulated in MCF-7 and MCF-12A cells (Table 1 and Supplementary Information S2). BUB3 and MAD2 proteins play important roles in the sensing microtubule spindle and kinetochore damage before the onset of mitosis [48], [49]. SPC25 and STMN1 are needed for the execution of mitotic events associated with kinetochore components and the formation of the mitotic spindle and progression through mitosis [49], [50]. Decreased expression of cyclin B and increased protein levels of cyclin B was observed in MDA-MB-231 cells while CDK1 gene expression was down regulated in all cell lines exposed to ESE-16 (Table 1, Table 2 and Supplementary Information S2). These results in addition to the morphological and cell cycle analyses from a previous study (Stander *et al*. 2011) further support the hypothesis that ESE-16 is an antimitotic compound that is able to interfere with spindle formation dynamics during mitosis [11].

The expression of mRNA for ferritin heavy polypeptide 1 (FTH1) and heme oxygenase 1 (HMOX1) were up regulated in response to ESE-16 exposure (Table 1). Both HMOX and FTH1 function in restoring iron metabolism and confers cytoprotection in response to cellular stress [51], [52]. While HMOX1 mRNA is strongly induced by ferric iron (Fe^3+^) [53], [54], [55], increased intracellular labile iron is an inducer of FTH1 expression [52]. Ferritin is known to be associated with microtubules and inhibitors of microtubule polymerization such as nocodazole inhibits these interactions [56]. Microtubule-associated oligomeric ferritin also contain higher amounts of iron than unbound monomeric ferritins and plays an important part in iron metabolism [56]. Interfering with microtubule dynamics may therefore contribute towards imbalances in iron metabolism and contribute towards iron-mediated cell death induction [57]. Further studies were carried out to test this possible link.

### Morphological Effects of ESE-16 on Breast Cancer Cells

**Figure 3:**
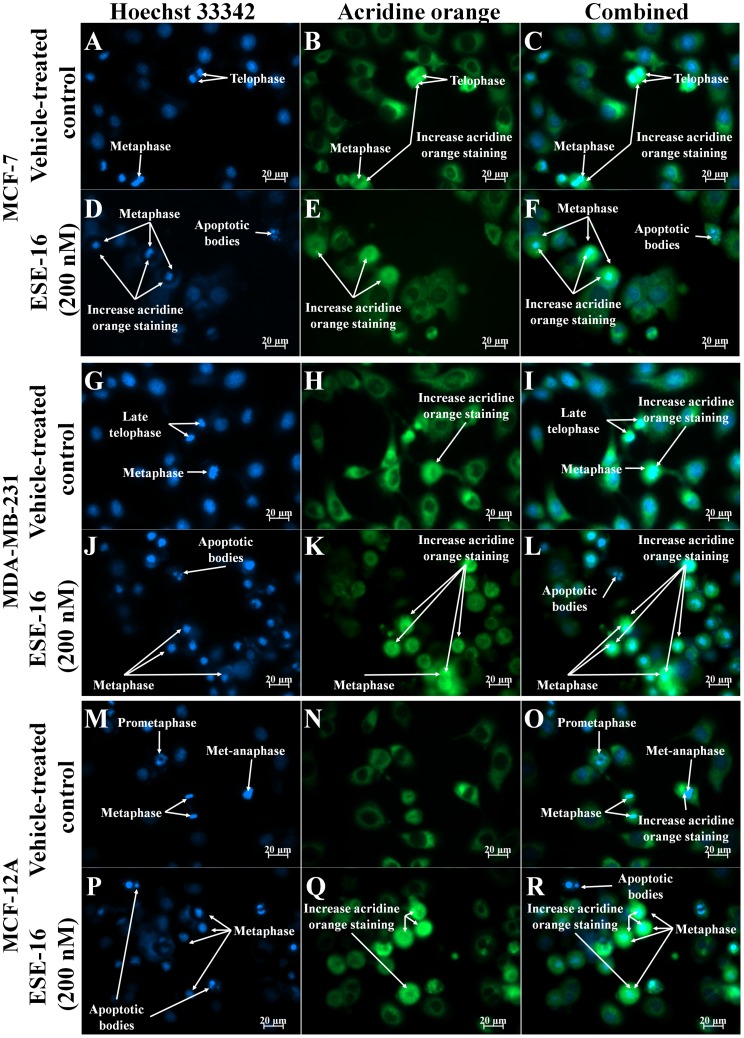
Hoechst 33342 and acridine orange-stained MCF-7, MDA-MB-231 and MCF-12A cells at 400× magnification after 24 h exposure. Cells completing cell division are observed in vehicle-treated cells (A–C, G–I and M–O) while an increase in the number of cells blocked in metaphase is observed in and ESE-16-treated (200 nM) cells (D–E, J–L and *P–*R). Acridine orange staining appears to be more concentrated in actively dividing cells in both treated and untreated cells.

**Figure 4:**
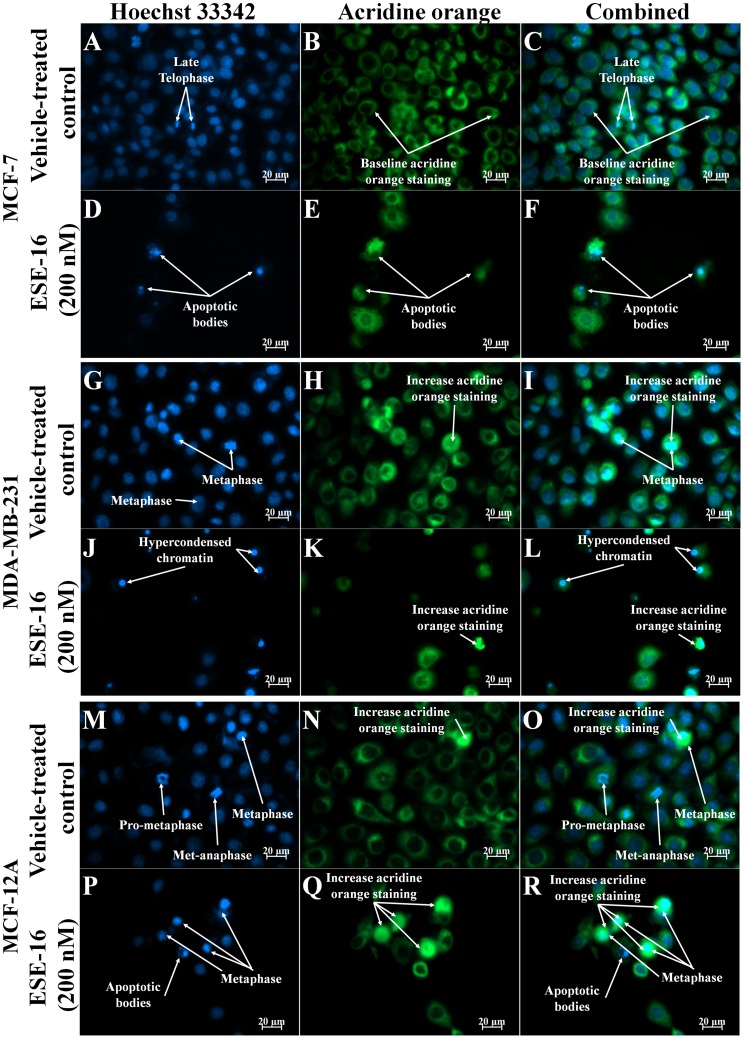
Hoechst 33342 and acridine orange-stained MCF-7, MDA-MB-231 and MCF-12A cells at 400× magnification after 48 h exposure. Vehicle-treated cells (A–C, G–I and M–O) in various stages of the cell cycle are observed. Formation of apoptotic bodies are observed in ESE-16-treated cells (G–I). An increase in the number of cells in metaphase is observed in ESE-16-treated MCF-12A (*P–*R) cells when compared to vehicle-treated MCF-12A cells (M–O). An increase in the formation of apoptotic bodies are observed in ESE-16-treated MCF-7 (D–E) and MDA-MB-231 (J–L) cells when compared to vehicle-treated cells (A–C and G–I).

Our previous study demonstrated that ESE-16 is an antimitotic compound that blocks MDA-MB-231 cells in the G_2_/M phase [11]. Gene and protein expression data in this study also demonstrated the differential expression of genes associated with mitosis. We therefore investigated the effects that ESE-16 has on nuclear as well as cytoplasmic morphology. The effects of ESE-16 (200 nM) on cell morphology was ascertained by means of fluorescent microscopy using acridine orange (green) and Hoechst 33345 (blue). Cells stained with Hoechst 33342 and acridine orange were observed at 400× magnification. Cells in various stages of cell division (including prometaphase, metaphase, met-anaphase, telophase) where observed in MCF-7 (Figure 3A, B, C and Figure 4 A, B, C), MDA-MB-231 (Figure 3G, H, I and Figure 4G, H, I) and MCF-12A (Figure 3M, N, O and Figure 4M, N, O) vehicle-treated cells after 24 h and 48 h. An increase in the number of cells blocked in metaphase was observed in ESE-16-treated MCF-7, MDA-MB-231 and MCF-12A cells after 24 h exposure (Figure 3D, E, F, J-K and P, Q, R), further supporting previous results indicating that ESE-16 is an antimitotic compound [11]. An increase in acridine orange staining was observed in cells blocked in metaphase in the treated samples. Acridine orange is a lysosomotrpic agent that fluoresces bright red in acidic lysosomal compartments and green in the cytosol. Increased green fluorescence may be indicative of lysosomal rupture as less OA is present in non-ruptured lysosomes and more in the cytosol.

After 48 h exposure, a marked increase in the formation of apoptotic bodies and hypercondensed chromatin was observed in ESE-16-treated MCF-7 and MDA-MB-231 cells (Figure 4 D, E, F, and, J-K). In MCF-12A-treated cells after 48 h exposure, an increase in the number of cells in metaphase is observed in ESE-16-treated cells (Figure 4P, Q, R) when compared to vehicle-treated cells (Figure 4M, N, O).

### Redox Status and Lysosomal Stability

**Figure 5:**
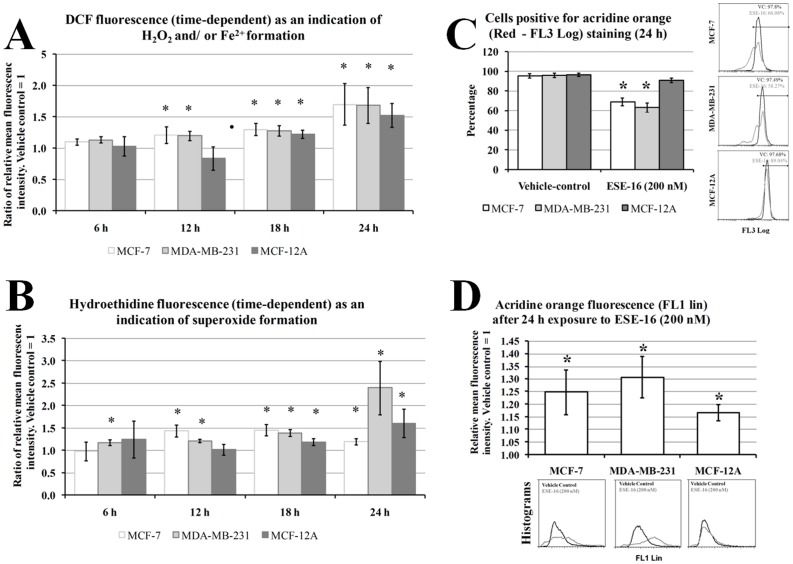
Effects of ESE-16 on ROS and lysosomal activity in MDA-MB-231, MCF-7 and MCF-12A cells after 24 h exposure. Relative mean fluorescence intensity of DCF (FL1) (A) hydroethidine (FL3) (B) in MCF-7, MDA-MB-231 and MCF-12A cells exposed to ESE-16 (200 nM). DCF and HE fluorescence increased over time, indicating increased formation of ROS. Red (C) and green (D) acridine orange (AO) fluorescence as indicators of lysosomal stability. Red fluorescent AO accumulates in healthy lysosomes while green fluorescent AO occurs in the cytosol. Decreased red red fluorescent AO and increased green AO fluorescence indicate increases in lysosomal rupture in ESE-16-treated cells. * and * indicate a P-value <0.05 between ESE-16- and vehicle-treated cells.

Various redox sensitive genes were differentially expressed, suggesting that ROS may play an active role in cell signaling of ESE-16-treated cells. In order to investigate whether increased amounts of hydrogen peroxide and/or ferrous iron (Fe^2+^) [25] and superoxide are produced in ESE-16-exposed (200 nM) cells compared to the control-exposed cells, flow cytometric analyses of MCF-7, MDA-MB-231 and MCF-12A cells loaded with the H_2_O_2_/Fe^2+^ sensitive fluorophore DCFDA and the superoxide sensitive probe hydroethidine. No difference in H_2_O_2_/Fe^2+^ formation was observed after 6 h exposure to ESE-16 (Figure 5A). The formation of H_2_O_2_/Fe^2+^ statistically significantly increased in ESE-16-exposed MCF-7, MDA-MB-231 and MCF-12A cells from 18 h to 24 h when compared to the vehicle-treated cells (Figure 5A).

Superoxide formation was statistically significantly increased from 6 h and continued to increase in ESE-16-treated MDA-MB-231cells when compared to the vehicle-treated control (Figure 5B). For MCF-7 cells superoxide formation increased from 12 h onwards in ESE-16-treated cells when compared to the vehicle-treated control (Figure 5B). For MCF-12A cells superoxide formation increased at 18 h and 24 h in ESE-16-treated cells when compared to the vehicle-treated control (Figure 5B). At 24 h, an increase superoxide formation was more pronounced in ESE-16-treated MDA-MB-231 cells when compared to MCF-7 and MCF-12A cells treated with ESE-16 (Figure 5B). The data suggest that ESE-16 is able to induce cellular changes that result on the increased formation of H_2_O_2_/Fe^2+^ and superoxide.

Karrlsson *et al.* (2010) have demonstrated that the DCF test is most likely an indication of lysosomal membrane permeability [25]. Since ESE-16 treatment resulted in increased DCF fluorescence, we wanted to see whether lysosomal membrane permeability was affected by ESE-16. Acridine orange was used in this assay. Red fluorescent AO accumulates in healthy lysosomes while green fluorescent AO occurs in the cytosol. The green and red fluorescence of acridine orange was quantified using flow cytometry in order to provide information about the stability of lysosomes in response to ESE-16 exposure. Red fluorescence decreased statistically significantly in MCF-7 and MDA-MB-231 cells exposed to ESE-16 (Figure 5C). A significant increase in green fluorescence was observed in ESE-16-treated MCF-7, MDA-MB-231 and MCF-12A cells (Figure 5D). These results, together with the fluorescent microscopy results indicate that lysosomal stability is negatively affected by ESE-16.

### Annexin V Externalization and Mitochondrial Membrane Potential

Gene and protein expression studies, as well as morphological investigations suggested that apoptosis is induced in response to ESE-16 exposure. Translocation of the membrane PS from the inner to the outer leaflet of the plasma membrane is normally one of the earliest indications of apoptosis. Annexin V is a 35–36 kDa, Ca^2+^-dependent, phospholipid binding protein with a high affinity for PS. A fluorescein isothiocyanate conjugated Annexin V was used to measure the translocation PS from the inner membrane to the outer membrane as an indication of apoptosis in ESE-16-treated (200 nM) MCF-7, MCF-12A and MDA-MB-231 cells.

**Figure 6:**
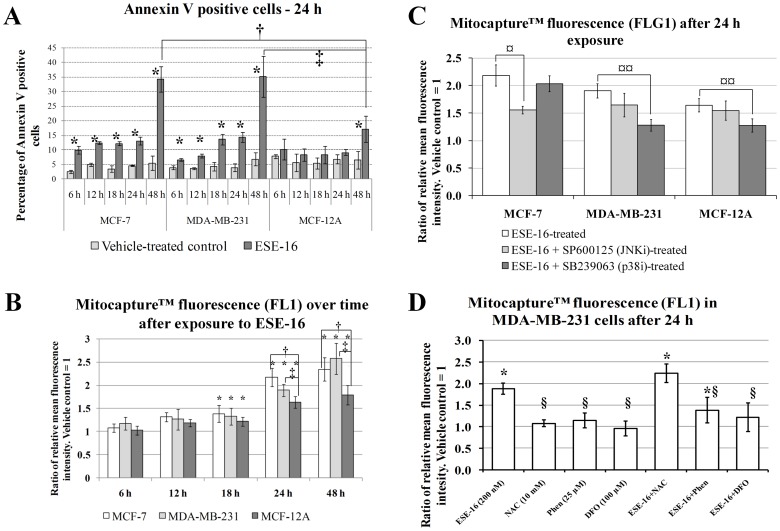
Effects of ESE-16 on phosphatidylserine externalization and mitochondrial membrane depolarization in MCF-7, MDA-MB-231 and MCF-12A cells over time. A) Measurement of phosphatidylserine externalization in MCF-7, MDA-MB-231 and MCF-12A cells. Apoptosis induction increases in a time-dependent manner in ESE-16-treated cells. The effect is more pronounced in MCF-7 and MDA-MB-231 cells. B) Comparison of differences in mitochondrial membrane depolarization in MCF-7, MDA-MB-231 and MCF-12A cells exposed to ESE-16 (200 nM) over time (6 h –48 h). Mitochondrial membrane depolarization increased in a time-dependent manner in ESE-17-treated cells. The effect is more pronounced in MCF-7 and MDA-MB-231 cells. C) The effects of JNK and p38 inhibitors on ESE-16-induced mitochondrial membrane depolarization. D) The effects of an antioxidant (NAC) and the iron chelators (DFO and 1,10-Phenanthroline) on ESE-16-induced mitochondrial membrane depolarization. * indicates a *P-*value <0.05 between vehicle-treated cells and ESE-16-treated (200 nM) cells. † indicates a *P-*value <0.05 between MCF-7 and MCF-12A cells exposed to ESE-16. ‡ indicates a *P-*value <0.05 between MDA-MB-231 and MCF-12A cells exposed to ESE-16. ¤ indicates a *P-*value <0.05 between ESE-16-treated cells and ESE-16-treated cells in combination with JNK inhibitor. ¤¤ indicates a *P-*value <0.05 between ESE-16-treated cells and ESE-16-treated cells in combination with p38 inhibitor. § indicates a *P-*value <0.05 between ESE-16-treated and other treated samples.

A gradual increase in PS externalization was observed in ESE-16-treated MCF-7 and MDA-MB-231 cells (Figure 6A). Also, a statistically significant increase in PS externalization was observed from 6 h onwards in ESE-16-treated MCF-7 and MDA-MB-231 cells when compared to the vehicle-treated control (Figure 6A). For MCF-12A cells, PS externalization was statistically significantly more after 48 h exposure to ESE-16 when compared to the vehicle-treated control (Figure 6A). After 24 h and 48 h exposure to ESE-16, PS externalization was statistically significantly more in MCF-7 and MDA-MB-231 cells when compared to MCF-12A cells (Figure 6A). These results indicate that ESE-16 is able to induce apoptosis in all the tested cell lines and that it is more pronounced in MCF-7 and MDA-MB-231 cells.

Mitochondrial membrane depolarization plays a key role in the intrinsic pathway of apoptosis induction. Mitochondrial membrane potential was analyzed using MitoCapture via flow cytometry. In apoptotic cells the reagent cannot aggregate in the mitochondria due to the altered membrane potential, and remains monomeric and in the cytoplasm generating a green fluorescence [26]. MCF-7, MDA-MB-231 and MCF-12A cells were exposed to 200 nM of ESE-16 and tested for any changes in mitochondrial membrane potential. Time-dependent studies on mitochondrial membrane were also carried out from 6 h to 48 h in MCF-7, MDA-MB-231 and MCF-12A cells exposed to ESE-16 (Figure 6B). A gradual increase in mitochondrial membrane depolarization was observed in ESE-16-treated cells when compared to the vehicle-treated control (Figure 6B). The effect was statistically significant after 18 h in all cell lines. After 24 h and 48 h, the effect was more pronounced in the MCF-7 and MDA-MB-231 cells when compared to the MCF-12A cells (Figure 6B).

Increased reactive species formation is able to induce the activation of mitogen-activated protein kinase 14 (p38α) and c-Jun N-terminal kinase (JNK) which in turn play a role in inducing apoptosis as well as mitochondrial membrane depolarization [58]. The effects of inhibiting the p38α and JNK1/2/3 kinases in inducing mitochondrial membrane were investigated in ESE-16-treated cells. SB239063 (15 µM [59]) and SP600125 (25 µM, [60]) were used to inhibit the p38α and JNK1/2/3 kinases respectively by incubating them with ESE-16 for 24 h. When treating MCF-7, MDA-MB-231 and MCF-12A cells only with the inhibitors, no apparent differences in mitochondrial membrane potential was observed when compared to the vehicle-treated controls (data not shown). In ESE-16-treated (200 nM) cells, there was a statistically significant increase in green fluorescence across all the cell lines after 24 h exposure, thereby indicating that mitochondrial membrane depolarization was induced in ESE-16-treated cells (Figure 6C). The JNK inhibitor had no effect in inhibiting or increasing this effect in MDA-MB-231 cells and MCF-12A cells. In MCF-7 cells however, the JNK inhibitor was able to reduce mitochondrial membrane depolarization in MCF-7 cells treated with ESE-16 (Figure 6C).

The p38α inhibitor had no effect in inhibiting or inducing mitochondrial membrane depolarization in MCF-7 cells (Figure 6C). However, the p38α inhibitor was able to reduce mitochondrial membrane depolarization in MDA-MB-231 cells and MCF-12A cells treated with ESE-16 (Figure 6C). While the respective inhibitors where unable to completely ablate the effects of ESE-16 on mitochondrial membrane potential, the data does suggest that JNK and p38α proteins play different roles in MCF-7, MDA-MB-231 and MCF-12A in inducing mitochondrial membrane depolarization in response to ESE-16 exposure.

Several reasons prompted us to investigate the effect that iron chelation had on modulating the effects of ESE-16 on mitochondrial membrane depolarization in MDA-MB-231 cells. Firstly, iron exist mainly within lysosomes and plays a major role in oxidant-induced cell death through mitochondrial membrane depolarization [61], [62]. Secondly, our results indicate that lysosomal stability is negatively affected by ESE-16. Release of intra-lysosomal content not only promotes oxidant-induced cell death due to the release of ferrous iron, it is also now established that it promotes oxidation DCF due to Fenton-type reactions [25]. Therefore, the increased DCF fluorescence may be due to lysosomal release of ferrous iron. Thirdly, gene expression studies indicated that several iron inducible genes were up regulated. We used both ferric (DFO) and ferrous iron chelators (Phen). DFO completely abolished mitochondrial membrane depolarization due ESE-16 exposure while Phen partially reduced mitochondrial membrane depolarization due to ESE-16 exposure (Figure 6D). Interestingly, NAC did not inhibit mitochondrial membrane depolarization (Figure 6D). One explanation for this result is that the hydrophilic nature of NAC hinders its entry into lysosomes and acts mainly in the cytosol, but is unable to inhibit the effects of ferrous iron release due to ruptured lysosomes. On the other hand, DFO and Phen are capable of sequestering the radical-forming ferrous iron in lysosomes, as well as in the cytosol before being released due to lysosomal instability [57].

### Active Caspase 3 and Caspase 7 Expression

Executioner caspases 3, 6 and 7 are responsible completing apoptotic cell death by destroying key components of cellular infrastructure and activate factors which damage the cell, and ultimately resulting in the morphological characteristics of apoptosis [63]. Dylight™ 488-conjugated secondary antibodies were used to bind to active caspase 3 and active caspase 7 primary antibodies that were fixed to vehicle-treated control, ESE-16-treated (200 nM) and Actinomycin D-treated (0.2 µg/ml) MCF-7, MDA-M-231 and MCF-12A cells after 24 h exposure. Actinomycin D was used as a possible control for apoptosis induction. Flow cytometry was employed to monitor fluorescence changes.

**Figure 7:**
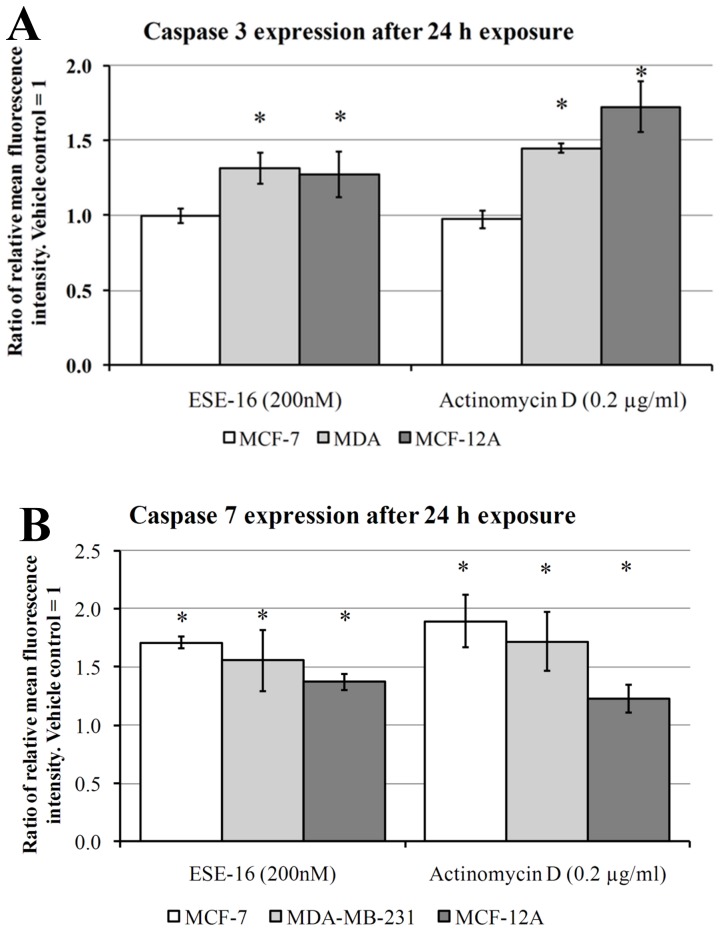
Effects of ESE-16 on caspase 3 and caspase 7 activity in MCF-7, MDA-MB-231 and MCF-12A cells after 24 h exposure. Relative fluorescence intensity for Dylight™ 488-conjugated secondary antibody bound to active caspase 3 (A) (FL1 log) or active caspase 7 (B) primary antibodies (FL1 log) after 24 h exposure to ESE-16 (200 nM). * indicates a *P-*value <0.05 between vehicle-treated cells and ESE-16-treated cells.

No difference in caspase 3 expression was observed in MCF-7 cells exposed to either Actinomycin D or ESE-16 treatment (Figure 7A). Caspase 3 protein expression was elevated in both ESE-16 and Actinomycin D-treated MDA-MB-231 and MCF-12A cells (Figure 7A). Caspase 7 protein expression was elevated in both ESE-16 and Actinomycin D-treated MCF-7, MDA-MB-231 and MCF-12A cells (Figure 7B). Together this data suggest that both caspase 3 and caspase 7 play a role in inducing apoptosis in ESE-16-treated cells. Caspase 3 does not appear to be expressed in MCF-7 cells, explaining why only caspase 7 is expressed in ESE-16-treated MCF-7 cells [64].

### Phosphorylation of Bcl-2 at Serine 70

Gene expression studies indicated that BBC3 expression was up regulated in all the cell lines in response to ESE-16 exposure. BBC3 protein expression is able to induce apoptosis by inhibiting the activity of various anti-apoptotic Bcl-2 proteins, including Bcl-2 [33]. This, together with the discovery that ESE-16 depolarizes mitochondrial membrane potential prompted us to investigate the phosphorylation status of Bcl-2. Bcl-2 is a key regulator of mitochondrial membrane potential and mitochondrial mediated apoptosis induction. An increase in the phosphorylation of Bcl-2 at serine 70 only lead to prevention of apoptosis induction while an increase in the multi-site phosphorylation status of Bcl-2 at serine 70, tryptophan 69 and serine 87 lead apoptosis induction [65]. Multi-site phosphorylation of Bcl-2 is associated with a G_2_/M block in MCF-7 and MDA-MB-231 cells [65]. Dephosphorylation of Bcl-2 at serine 70 is also associated with apoptosis [65]. Also, an overall decrease in the protein expression of Bcl-2 is pro-apoptotic [65].

Flow cytometry was employed to study the phosphorylation status of Bcl-2 at Ser 70 (pBcl-2 (ser70)) in the MCF-7, MDA-MB-231 and MCF-12A cell lines after 24 h exposure to ESE-16 (200 nM) compared to the vehicle-treated control. The expression levels of pBcl-2 (ser70) were also monitored in ESE-16 (200 nM) MCF-7, MDA-MB-231 and MCF-12A cells. The FlowCellect Bcl-2 Activation Dual Detection Kit (Millipore) uses two antibodies to measure the abundance of Bcl-2 protein expression generally and the abundance of Bcl-2 phosphorylated at Ser 70 specifically (FL3 Log).

**Figure 8:**
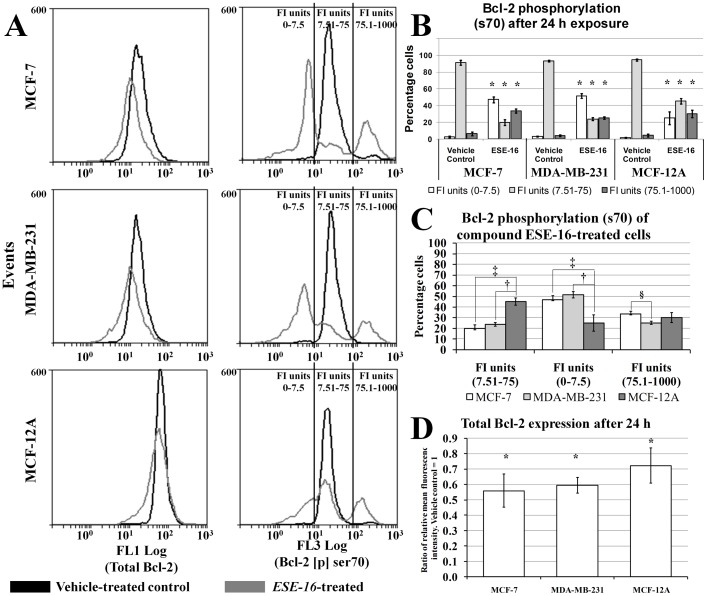
Effects of ESE-16 on Bcl-2 phosphorylation and expression in MCF-7, MDA-MB-231 and MCF-12A cells after 24 h exposure. A) Flow cytometry histograms of total Bcl-2 content (FL1 Log) and Bcl-2 phosphorylated at Ser70 (FL3 log) in MCF-7, MDA-MB-231 and MCF-12A cells after 24 h exposure to ESE-16 (200 nM). Fluorescence Intensity units = FI units. B) Bar-chart demonstrating the distribution of fluorescence intensity (FI) units of Bcl-2 (Ser 70) (FL3 Log) labeled MCF-7, MBA-MB-231 and MCF-12A cells after 24 h exposure to ESE-16 (200 nM). C) Comparison of differences in distribution of fluorescence intensity (FI) units of Bcl-2 (Ser 70) (FL3 Log) labeled MCF-7, MDA-MB-231 and MCF-12A cells after 24 h exposure to ESE-16 (200 nM). D) B) Total Bcl-2 expression after 24 h for MCF-7, MDA-MB-231 and MCF-12A cells after 24 h exposure to ESE-16 (200 nM).

ESE-16 exposure resulted in a decrease in the total amount of Bcl-2 protein (FL1 Log) when compared to the vehicle-treated cells in all cell lines (Figures 8A and 8D, Supplementary Information S3). For the FL3 log fluorescent channel, a fluorescence intensity (FI) unit range of 7.51–75 corresponds to the phosphorylation status of pBcl-2 (ser70) for >90% of cells in the vehicle-treated samples in all cell lines (Figures 8A 8B, Supplementary Information S3). From this, it was concluded that an increase in the number of cells with an FI unit range greater than 75 would constitute an example of a population of cells with increased pBcl-2 (ser70) expression when compared to the vehicle-treated control. Also, an increase in the number of cells with an FI unit range less than 7.5 would constitute an example of a population of cells with decreased pBcl-2 (ser70) expression when compared to the vehicle-treated control. ESE-16 treatment resulted in a statistically significant decrease in the number of cells with an FL3 Log FI unit range of 7.51–75 and an increase in the number of cells with an FL3 Log FI unit range of less than 7.5 and more than 75 (Figure 8B). The decrease in the amount of cells with an FL3 Log FI unit range of 7.51–75 was statistically significantly more in ESE-16-treated MCF-7 and MDA-MB-231 cells when compared to MCF-12A cells (Figure 8C). Also, the amount of cells with an FL3 Log FI unit range of less than 7.5 was statistically significantly more in ESE-16-treated MCF-7 and MDA-MB-231 cells when compared to MCF-12A cells (Figure 8C). The dephosphorylation (increase in number of cells with an FL3 Log FI unit range of less than 7.5) of pBcl-2 (ser70), as well as the overall reduction in Bcl-2 expression suggests that ESE-16 is able to abrogate the balance of Bcl-2 phosphorylation as well as Bcl-2 expression in a manner that promotes apoptosis via intrinsic pathways.

### Conclusions

The present study demonstrates that ESE-16 is more selective at inhibiting the activity of the CAIX mimic when compared to the wild-type CAII. The inhibition is 1.45 times more selective and occurs in the nanomolar range, confirming the results from the docking studies from previous studies [11]. *In vitro* studies on MDA-MB-231 cells suggest that ESE-16 is able to abrogate extracellular acidification via CAIX expression. High resolution (400x) fluorescence microscopy using Hoechst 33342 and acridine orange fluorescent stains revealed that cells blocked in metaphase have an increased propensity to be stained with acridine orange. Increased acridine orange staining is an indication of lysosomal instability and lysosomal rupture. Flow cytometry using acridine orange confirmed that lysosomal rupture is increased in ESE-16-treated cells and was more pronounced in the tumorigenic MCF-7 and metastatic MDA-MB-231 cells when compared to MCF-12A cells.

Gene and proteinarray expression studies provided valuable information that was used to guide further studies. Differential expression of genes associated with cell death, as well as redox- and iron-sensitive genes implicated apoptosis and redox signaling as possible mechanism of growth inhibition due to ESE-16 treatment. Cell death via apoptosis was confirmed via Annexin V flow cytometry assays and caspase 3 and 7 also appear to be activated in response to ESE-16. Flow cytometry analyses also confirmed that mitochondrial membrane depolarization is increased in response to ESE-16 treatment, indicating that the mitochondrial pathway is a likely player in inducing caspase-mediated cell death.

The phosphorylation status of Bcl-2 plays a key role in regulating mitochondrial membrane potential and mitochondrial mediated apoptosis induction. Flow cytometry analyses using an anti-Bcl-2 antibody and an antibody that is specific for phosphorylated Bcl-2 at Ser 70 demonstrated that ESE-16 is able to alter the total Bcl-2 content as well as Bcl-2 phosphorylation (Ser 70) dynamics in cells in the MCF-7, MDA-MB-231 and MCF-12A cell lines. The effects were more pronounced in the MCF-7 and MDA-MB-231 cells when compared to the MCF-12A cells.

It was observed that superoxide formation increased and an increase in DCF fluorescence was indicative of hydrogen peroxide and/or ferrous iron. Superoxide can reduce ferric iron to ferrous iron and ferrous iron together with hydrogen peroxide participate in Fenton reactions that can cause ROS formation and activate important stress signaling pathways [66]. Excessive ROS is able to activate the ASK1–Trx signalosome, which in turn is able to activate downstream activation of stress activated protein kinases (SAPKs), JNK1 and p38α, which may affect the mitochondrial polarization status. The results indicate JNK and p38 pathways are differentially activated MCF-7 and MDA-MBA-231 cells in response to ESE-16 treatment.

**Figure 9:**
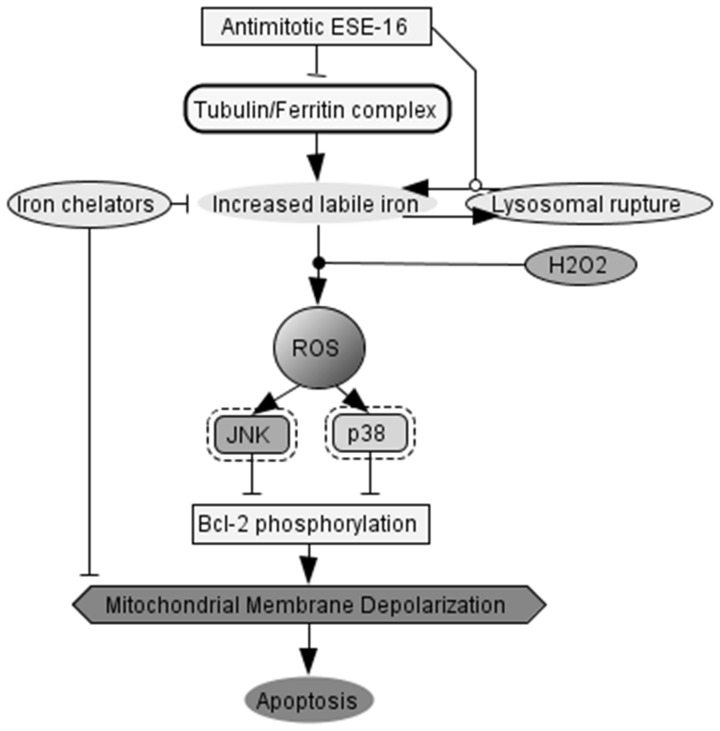
Hypothesis for the mechanism of action of ESE-16 on MCF-7 cells. ESE-16 increases labile iron due to lysosomal rupture and possibly interfering with tubulin/ferritin complexes in actively dividing cells. This in turn contributes towards ROS signaling and activates stress-activated kinases. ESE-16 exposure ultimately leads to the abrogation of Bcl-2 phosporylation and induces apoptosis via mitochondrial membrane depolarization.

Finally, the observation of lysosomal rupture, oxidation of DCF due to Fenton-type reactions and gene expression changes in several iron inducible genes prompted us to investigate the effect that iron chelators had on modulating the effects ESE-16. Iron chelators abolished mitochondrial membrane depolarization in MDA-MB-231 cells treated with ESE-16. These results suggest that labile iron plays an important role in mediating cell death associated with ESE-16 exposure. Shpyleva *et al.* (2011) discovered that changes in intracellular ferritin levels correlates well with more malignant breast cancer cells [67]. More malignant MDA-MB-231 and MCF-7 cells may therefore be more sensitive towards iron-mediated cell death induction [57]. The fact that inhibitors of microtubule polymerization such as nocodazole (and possibly ESE-16) lead to perturbations in iron metabolism [56] provides a possible explanation for the apparent selectivity of ESE-16 towards the more malignant MDA-MB-231 and MCF-7 cells when compared to the non-malignant MCF-12A cells (Figure 9). Future studies are underway to determine the exact nature between microtubules, iron metabolism, cell death via lysosomal rupture and antimitotic compounds.

To conclude, the data from this study yielded valuable information about the mechanism of action of ESE-16 on various breast cell lines. Lysosomal rupture and apoptosis induction via mitochondrial membrane depolarization due to alterations in Bcl-2 phosphorylation provides a basis for future studies. Also, data from the global gene and protein expression experiments suggest that further studies (that could not be pursued in the present study) focusing on the role of the unfolded protein response pathway in response to ESE-16 treatment are warranted.

## Supporting Information

Supporting Information S1 **Detailed explanation of methods.**
(DOCX)

Supporting Information S2 **Extensive list of differentially expressed genes (adjusted** ***P-*** **value <0.05) mapped to functional cellular pathways in MCF-7, MDA-MB-231 and MCF12A cells exposed to ESE-15-one (140 nM), ESE-15-ol (50 nM), and ESE-16 (200 nM) for 24 h.**
(DOCX)

Supporting Information S3 **Bcl-2 flow cytometry data.** Percentage of cells in the FI unit ranges of 0–7.5, 7.51–75 and 75.1–1000 as an indication of the quantity of Bcl-2 (Ser 70) phosphorylation per cell.
(DOCX)
