# Correction: Signaling Pathways of ESE-16, an Antimitotic and Anticarbonic Anhydrase Estradiol Analog, in Breast Cancer Cells

**DOI:** 10.1371/annotation/6afd5e63-46d6-42a1-b661-6688a04bfd16

**Published:** 2013-10-04

**Authors:** Barend Andre Stander, Fourie Joubert, Chingkuang Tu, Katherine H. Sippel, Robert McKenna, Annie Margaretha Joubert

There was an error in the affiliation for author Annie Margaretha Joubert. Her affiliation should be Department of Physiology, University of Pretoria, Pretoria, Gauteng, South Africa. 

