# Supplementary material for: Signaling Pathways of ESE-16, an Antimitotic and Anticarbonic Anhydrase Estradiol Analog, in Breast Cancer Cells
Source: PLoS One. 2013 Jan 31;8(1):e53853. doi: 10.1371/journal.pone.0053853 (PMC3561402; doi:10.1371/journal.pone.0053853)
Supplement: Supporting Information S1 — Detailed explanation of methods. (DOCX) [file pone.0053853.s001.docx]

Supplementary Information S1

**Methods**

**Gene expression analysis: Complimentary RNA microarray**

**I) RNA extraction**

Exponentially growing MCF-12A, MCF-7 and MDA-MB-231 cells was seeded at 750 000 cells per 25cm^2^ flask. After 24 h attachment the medium was discarded and the cells exposed to 200 nM of ESE-16 and incubated for 24 h. After exposure, the cells were washed twice with PBS and lysed by adding 1 mL RLT buffer (RNeasy Mini Kit) to each flask and the flask was decanted until all the cells were lysed. The lysate was pipetted into Qiashredder columns and centrifuged for 2 min at 9000xg. The flow-through was collected and 1 volume of 70% ethanol was added and gently mixed. This solution was divided into Qiagen Plant Mini Kit columns (700 µL per column) and centrifuged for 15 s at 9000xg. The flow-through was discarded. 350 µL RW1 buffer was added to each column in order to wash the column. The column was centrifuged for 15 s at 9000xg. A DNase mixture was prepared utilizing Qiagen’s RNase-free DNase Set, by adding 70 µL RDD buffer to every 10 µL DNase1. 80 µL of this mixture was added in the middle of each column and left at room temperature for 15 min. Another 350 µL RW1 buffer was added and the column was centrifuged for 15 s at 9000xg. Flow-through was discarded and columns transferred to new eppendorf tubes. A series of washing steps followed. 500 µL RPE buffer was added to each tube and centrifuged for 15 s at 12000xg, discarding the flow-through afterwards and replacing the eppendorf tubes. This step was repeated. The column was the then centrifuged at 9000xg for 1 min. To elute the total RNA from the column 50 µL RNase-free water was added to the column and centrifuged for 1 min at 9000xg. The total RNA was suspended in 50 µL RNase-free water and was ready to be quantified with the Nanodrop and tested for integrity by means of electrophoresis.

**II) RNA integrity**

0.6g agarose powder was dissolved in 40ml RNase free water (1.5% gel). 6ml 10x
3-(N-morpholino)propanesulfonic acid (MOPS), 3.4 ml formaldehyde and 14 ml RNase free water was added. A sample mix to be loaded in the well was prepared by adding 3µg total RNA (±8µL), 2µL tracking dye (50% glycerol, 100mM Na_2_EDTA, pH 8.0, 1% SDS, 0.1% bromophenol blue 0.1%) and 1µL ethidium bromide (0.5 µg/mL final concentration). The mix was heated for 15min at 55ºC and rapidly chilled on ice afterwards. 15µL of each sample was pipetted into a well of the agarose-formaldehyde RNA gel and electrophoresis was conducted at 80mV for 45min.

**III) Labelled cRNA synthesis**

Agilent’s Low RNA Input Fluorescent Linear Amplification Kit was used to generate fluorescently labeled cRNA. Labeled cRNA was produced using the T7 polymerase and purification of labeled cRNA was conducted using Qiagen’s RNeasy Mini kits. For each sample, 2000 ng of total or polyA RNA was added to a 1.5 mL microcentrifuge tube and filled with RNase free water to a final volume of 8.3 µL or less.

Spike A Mix (2.0 µL) containing the positive control mRNA for the cyanine 3-CTP dye reactions and the Spike B Mix (2.0 µL) containing the positive control mRNA for the cyanine 5-CTP dye reactions were added to their respective samples containing the extracted total RNA from either the vehicle-treated controls or the compound-treated experiments. 1.2 µL of T7 Promoter Primer (from the Agilent Low RNA Input Linear Amplification Kit PLUS, Two-Color) was added to each sample and the primer and the template were denatured by incubating the reaction at 65°C in a circulating water bath for 10 minutes. After 10 mins the reactions were placed on ice and incubated for 5 minutes.

For each sample 4 µL 5X First Strand Buffer, 2 µL 0.1 M Dithiothreitol (DTT), 1 µL 10 mM dNTP mix (10mM dATP, dCTP, dGTP, dTTP), 1 µL Moloney Murine Leukemia Virus Reverse Transcriptase (MMLV-RT) and 0.5 µL RNase inhibitor (RNaseOUT) was added and incubated at 40°C in a circulating water bath for 2 hours. After 3 hours the samples were incubated at 65°C in a circulating water bath for 15 minutes after which it was incubated on ice for 5 minutes.

The samples were briefly centrifuged to drive down any precipitation. For each sample 15.3 µL nuclease-free water, 20 µL of 4X Transcription Buffer, 6 µL of 0.1 M DTT, 8 µL of NTP mix, 0.5 µL 50% Polyethylene glycol (PEG) 6.4 RNaseOUT, 0.6 µL Inorganic pyrophosphatase, 0.8 µL T7 RNA Polymerase and 2.4 µL of either Cyanine 3-CTP or cyanine 5-CTP. The samples were incubated at 40°C in a circulating water bath for 2 hours.

The labeled cRNA was purified using Qiagen’s RNeasy mini spin columns. To each sample, 20 µL of nuclease-free, 350 µL of Buffer RLT and 250 µL of ethanol (100% purity) were added and mixed thoroughly by pipetting. This solution was pipette onto the RNeasy mini spin columns columns (700 µL per column) and centrifuged for 15 s at 9000xg. The flow-through was discarded and 500 µL RPE buffer was added to each tube and centrifuged for 15 s at 12000xg, discarding the flow-through afterwards and replacing the eppendorf tubes. This step was repeated. The column was the then centrifuged at 9000xg for 1 min without anything added to column in order to remove all residual liquid. In order to elute the total RNA from the column 35 µL RNase-free water was added to the column and centrifuged for 1min at 9000xg. The labeled cRNA was suspended in 35 µL RNase-free water and was quantified with the Nanodrop.

**V) Hybridization of Cy-dye labeled cRNA**

Cleaned cRNA was hybridized to Agilent Human 1A (V2) oligonucleotide 44K microarray slides according to the manufactures guidelines using Agilent’s 2x GEx Hybridization Buffer HI-RPM in Agilent’s SureHyb chambers. A hybridization mixture was prepared by adding equal amounts of Cy-3 and Cy-5 labeled cRNA (825 ng), 11 µL 10X blocking Agent, 2.2 of 25x Fragmentation Buffer to a final volume of to a final volume of 55 µL. The samples were incubated at 60°C for exactly 30 minutes to fragment the RNA. After 30 min, 55 mL of the 2x Hybridization Buffer was added to each sample. The backing slides were placed in Agilent’s microarray hybridization chambers and the above prepared solution was pipette (100 µL) onto each grid (four grids on one slide).

The assembled slide chamber wes placed in Agilent’s hybridization ovens set to 65°C and
10 rpm. The samples were hybridized at for 17 hours. Afterwards the slides were washed twice for 1 min in Falcon tubes containing Agilent’s Gene Expression Wash Buffer 1 at room temperature and once in Falcon tubes containing Agilent’s Gene Expression Wash Buffer 2 at 37°C for 1 min. The slide was transferred to acetonitrile for 1 min and then subsequently to Agilent’s Stabilization and Drying Solution to help prevent ozone bleaching of the Cy-fluorochromes. The slides were gently removed from the Stabilization and Drying Solution and scanned immediately.

**VI) Scanning of Agilent microarray slides**

Slides were scanned with the Axon Genepix 4000B Scanner (Molecular Devices, USA) provided by the African Centre of Gene Technology (ACGT) Microarray Facility at the University of Pretoria.

**VII) Spotfinding**

Spotfinding was performed using Genepix Pro 6.1 (Molecular Devices Corporation, Sunnyvale, California USA). GenePix Pro 6.1 uses a set of proprietary feature-finding algorithms to find circular features. Every pixel in a region around the feature is examined by a local alignment algorithm and assigned to feature or background. Global alignment algorithms determine the translation, rotation and skew of blocks of features. Saturated spots, spots with an uneven background, non-uniform spots and spots with a low intensity vs back ground ratio were removed from further analysis by excluding the spots that satisfied the following parameters. The excluded spots were assigned as “Bad”.

**Saturated spots:**

[F532 % Sat.] > 30 And

[Ratio of Means (635/532)] > 0.75 Or

[F635 % Sat.] > 30 And

[Ratio of Means (635/532)] < 1.3333

**Spots with an uneven background:**

([B635 Mean] > (1.5*[B635 Median]) Or

[B532 Mean] > (1.5*[B532 Median])) And

([B635 Median] > 40 Or

[B532 Median] > 40)

**Non-uniform spots:**

[Ratio of Medians (635/532)] > (4.0*[Rgn Ratio (635/532)]) Or

[Ratio of Medians (635/532)] < (0.25*[Rgn Ratio (635/532)])

**Low intensity vs back ground ratio:**

[% > B635+2SD] < 10 Or [% > B532+2SD] < 10

**VIII) Limma statistical analysis**

Statistical analysis after spotfinding was conducted using Limma with the LimmaGUI interface [[1](#_ENREF_1),[2](#_ENREF_2)]. Background correction was done with the normal+exponential (Normexp) convolution model to observed intensities. The normal part represents the background and the exponential represents the signal intensities [[2](#_ENREF_2)]. The Normexp offset value was set to 25. A value of 25 maximized the df.prior. df.prior is the numeric vector giving empirical Bayes estimated degrees of freedom associated with s2.prior for each gene. s2.post is the numeric vector giving posterior residual variances. A maximized df.prior is optimal and will allow for greater power to detect differentially expressed genes [[2](#_ENREF_2)]. Spot quality weighting was performed and Genepix Flag weightings that were flagged as “Bad” were excluded from further analysis. Normalization within arrays was performed to remove dye-bias at higher and lower intensities by normalizing M-values (log-ratios) with the Global Loess method [[3](#_ENREF_3)]. The M-value (M) represents a log2-fold change between two or more experimental conditions. The A-value (A) is the average log2-expression level for a gene across all the arrays and channels in the experiment.

The M-value is calculated as follows:

M = log2(Cy5)/Cy3) (Cy5/Cy3 are the normalized emission intensities of the spot)

The A-value is calculated as follows:

A = (log2(Cy5)*Cy3))/2

Aquantile normalization between arrays was performed in order to normalize expression intensities so that the intensities or log-ratios have similar distributions across a series of arrays [[3](#_ENREF_3)]. Aquantile normalization ensures that the A-values (average intensities) have the same empirical distribution across arrays leaving the M-values (log-ratios) unchanged [[3](#_ENREF_3)]. The Least squares linear model fit method was employed and the *P-*values were adjusted for multiple testing utilizing the Benjamini and Hochberg’s step-up method for controlling the false discovery rate [[4](#_ENREF_4)]. Genes that had a *P*-value of less than 0.05 were considered statistically significantly differentially expressed and were included in further analyses.

**IX) Gene expression analysis**

Biological interpretation and functional analysis of gene lists were performed by mapping differentially expressed genes to biochemical pathways and Gene Ontology (GO) categories using Gene Annotation Co-occurrence Discovery (GENECODIS) [[5](#_ENREF_5)]. GENECODIS is a web-based tool for finding sets of biological annotations that frequently appear together and are significant in a set of genes [[5](#_ENREF_5)]. In order to determine common genes that were affected by the compounds *­* between cell lines as well as the different compounds, differentially expressed gene lists were compared utilizing GeneVenn [[6](#_ENREF_6)]. GeneVenn is a simple, web-based application creating Venn diagrams from two or three gene lists.

**Protein expression analysis: Protein microarray**

**I) Protein extraction and concentration determination**

Exponentially growing MCF-7 and MDA-MB-231 cells were seeded at 2 000 000 cells per 75 cm^2^ flask. After 24 h attachment the medium was discarded and the cells exposed 200 nM of ESE-16 and incubated for 24 h. After exposure, cells were washed 4 times with PBS and then trypsinized. The cells were collected and centrifuged. The supernatant was removed and the pellet was frozen at -80 °C and then weighed. 20 µL of Extraction/Labeling Buffer was added for every 1mg of cells and incubated for 10 min. The sample was then centrifuged at 10,000 x g for 30 min at 4 °C. Afterwards, the supernantant was transferred to a new 1.5 mL tube and the protein concentration was measured with BCA (Pierce’s BCA Protein Assay Reagent Kit). Bovine serum albumin from the kit was used to make various standard concentrations of protein ranging from 0 mg/mL to 2.5 mg/mL. The BCA kit consists of two parts. Reagent A contains sodium carbonate, sodium bicarbonate, bicinchoninic acid and sodium tartrate in 0.1 M sodium hydroxide. Reagent B is 4% cupric sulfate. A working solution was freshly made by mixing 50 parts of Reagent A and 1 part of Reagent B. 10 µL of each protein sample was pipetted into a 96-well plate and mixed with 200 µL of the working solution. The samples were covered with foil and incubated for 30 min at 37 °C. The absorbance at 570 nM of each sample was measured with an EL_x_800 Universal Microplate Reader from Bio-Tek Instruments Inc. (Vermont, United States of America). The linear relationship between the absorbance and the standard concentrations was used to determine the concentration of the protein in the control and exposed cell samples. Each cell sample was diluted with Extraction/Labeling Buffer to yield a final concentration of 1.1 mg/mL.

**II) Protein labeling**

Two antibody array slides were used for each cell line. There are two replicates of each protein an antibody array slide. Therefore, two biological replicates and two technical replicates provided an *n* number of 4. For the first biological replicate, the control was labeled with Cy3 dye (green) and the experiment was labeled with Cy5 dye. For the second biological replicate, the control was labeled with Cy5 dye (green) and the experiment was labeled with Cy3 dye. This two-slide dye-swop methodology is used to remove any dye bias that may result due to differences in Cy-dye incorporation. Fresh Cy3 and Cy5 mono-Reactive Dyes were reconstituted with 110 µL Extraction/Labeling Buffer. 94 µL of the protein samples (1.1 mg/mL) was mixed with 6 µL of the reconstituted Cy-dyes and incubate at 4°C for 90 min in the dark. The samples were mixed by inversion every 20 min. After 90 min, 4 µL Blocking Buffer was added and further incubated for 30 min at 4°C in the dark. The samples were mixed by inversion every 10 min.

**III) Removal of Unbound Cy-Dye and determination of Cy-dye incorporation**

Zeba Spin Desalting Columns (7K MWCO) were used to desalt the samples and remove any unreacted Cy-dye. The storage solution was removed by centrifugation and the protein samples (±104 µL) was loaded onto the desalting columns and centrifuged at 1,500×g for 2 minutes. Bovine serum albumin from the BCA kit was used to make various standard concentrations of protein ranging from 0 mg/mL to 0.5 mg/mL and the protein concentration (${ProtConc}_{mg/ml}$) was measured as described above. Cy3 absorbance ($A552$) and Cy5 absorbance ($A650$) were measured with a Nanodrop. Incorporation of Cy3 and Cy5 dyes into protein was calculated as follows. For Cy3, a molar extension coefficient ($\varepsilon552$) of 150,000 M^–1^.cm^–1^ was used as suggested. The average molecular weight of protein was assumed to be 60,000 Da (as suggested by the manual).

The Cy3 concentration (${Cy3Conc}_{\mu M}$) in the sample was calculated as:

$${Cy3Conc}_{\mu M}=\left( \frac{A552}{\varepsilon552} \right)\times{10}^{6}$$

The protein concentration ($ProtConc{552}_{\mu M}$) in the sample was calculated as:

$$ProtConc{552}_{\mu M}=\frac{{ProtConc552}_{mg/ml}}{60000 Da} \times{10}^{6}$$

For Cy5, a molar extension coefficient ($\varepsilon650$) of 250,000 M^–1^.cm^–1^ was used as suggested. The average molecular weight of protein was assumed to be 60,000 Da (as suggested by the manual).

The Cy5 concentration (${Cy5Conc}_{\mu M}$) in the sample was calculated as:

$${Cy5Conc}_{\mu M}=\left( \frac{A650}{\varepsilon650} \right)\times{10}^{6}$$

The protein concentration (ProtConc650_µM) in the sample was calculated as:

$$ProtConc{650}_{\mu M}=\frac{{ProtConc650}_{mg/ml}}{60000 Da} \times{10}^{6}$$

The following ratios yielded information regarding the average number of coupled dye molecules for the respective dyes:

$$\frac{{Cy3Conc}_{\mu M}}{{ProtConc552}_{\mu M}} and \frac{{Cy5Conc}_{\mu M}}{{ProtConc650}_{\mu M}}$$

A ratio of 2-4 for each of these ratios was considered sufficient to proceed to hybridization.

**V) Hybridization of Cy-dye labeled protein**

100 µg of Cy3-labeled vehicle-control protein and 100 µg of Cy5-labeled C19-treated protein was added together for slide 1 (Slide 1 mix). 100 µg of Cy3-labeled C19-treated protein and 100 µg of Cy5-labeled vehicle-control protein were added together for slide 2 (Slide 2 mix).

For each slide, 45 mL of was prepared by mixing 4.5 mL Background Reduce and 40.5 mL Stock Incubation Buffer. 5 mL of Incubation Buffer was added to each incubation chamber as well as wash chamber. Thereafter, 20 µg of Slide 1 mix was added to the incubation chambers containing the 5 mL of Incubation Buffer for slide 1 and mixed for 10 min at room temperature in the dark. 20 µg of Slide 2 mix was added to the incubation chambers containing the 5 mL of Incubation Buffer and mixed for 10 min at room temperature in the dark.

The slides were prepared by decanting the storage buffer from the storage vial if the slides into a waste beaker. 30 mL of Stock Incubation Buffer was added into the storage vial and slowly inverted for 10 min. The Stock Incubation Buffer was decanted and fresh Stock Incubation Buffer (20 mL) was added to the storage vial. The storage vial was slowly inverted for 10 min. After the washing of the slides, a slide was placed into each incubation tray and incubated at room temperature for 40 min while slowly rocking the incubation trays.

After 40 min, 5 mL of Wash Buffer A was added to the wash chambers already containing Incubation Buffer. The slides were gently transferred to the wash chamber and incubated for 5 min. The buffer from each wash chamber was gently removed and replaced with 5 mL of Wash Buffer B and incubated for 5 min at room temperature. Wash buffer B was then removed and replaced with 5 mL of Wash Buffer C and incubated at room temperature for 5 min. The slides were then transferred to 50 mL tubes containing deionized water and the transferred again to an empty 50 ml tube. The slides were then dried by centrifuging the tubes for 5 min at 1000xg at room temperature.

Slides were scanned with the Axon Genepix 4000B Scanner (Molecular Devices, USA) provided by the African Centre of Gene Technology (ACGT) Microarray Facility at the University of Pretoria.

**VI) Spotfinding**

Spotfinding was performed using Genepix Pro 6.1 (Molecular Devices Corporation, Sunnyvale, California USA). GenePix Pro 6.1 uses a set of proprietary feature-finding algorithms to find circular features. Saturated spots, spots with an uneven background, non-uniform spots and spots with a low intensity vs back ground ratio were removed from further analysis by assigning them as “Bad”.

**VII) Statistical analysis**

Clontech provides an Excel spreadsheet to determine an Internally Normalized Ratio (INR). The aim of the INR is to control for differences in labeling efficiency. An INR is calculated for each antibody antigen pair on the microarray.

The INR is calculated as follows ($VC$ = spot of vehicle-treated control, $C19$ = spot of C19-treated experiment):

$$Cy5=Cy5 mean intensity-Cy5 background$$

$$Cy3=Cy3 mean intensity-Cy3 background$$

$$Ratio 1= \frac{Cy5\left( VC:Slide 1, technial repliate 1 \right) \times Cy3(VC:Slide 2, technial repliate 1)}{Cy3\left( C19:Slide 1, technial repliate 1 \right) \times Cy5(C19:Slide 2, technial repliate 1)}$$

$$Ratio 2= \frac{Cy5\left( VC:Slide 1, technial repliate 2 \right) \times Cy3(VC:Slide 2, technial repliate 2)}{Cy3\left( C19:Slide 1, technial repliate 2 \right) \times Cy5(C19:Slide 2, technial repliate 2)}$$

INR = $\sqrt{\frac{Ratio 1+Ratio 2}{2}}$

This method relies on the background subtraction method of Genepix and it does not remove dye-bias at higher and lower intensities through normalization. Therefore, it was decided to use Limma with the LimmaGUI interface for statistical analyses [[1](#_ENREF_1),[2](#_ENREF_2)]. Background correction was done via log-linear interpolation [[7](#_ENREF_7),[8](#_ENREF_8)]. Spot quality weighting was performed and Genepix Flag weightings that were flagged as “Bad” were excluded from further analysis. Normalization within arrays was performed to remove dye-bias at higher and lower intensities by normalizing M-values (log-ratios) with the Global Loess method [[3](#_ENREF_3)]. Aquantile normalization between arrays was used [[3](#_ENREF_3)].

The *M-value* (M) represents a log_2_-fold change between the Cy5 and Cy3 channel intensities:

$$M={Log}_{2}(\frac{Cy5}{Cy3})$$

where $(\frac{Cy5}{Cy3})$ are the normalized emission intensities of a spot.

The Average *M-value* of a particular protein was calculated as follows:

$$M_{Technical replicate 1}= {Log}_{2}(\frac{Cy5\left( VC:Slide 1, technial repliate 1 \right) \times Cy3\left( VC:Slide 2, technial repliate 1 \right)}{Cy3\left( C19:Slide 1, technial repliate 1 \right) \times Cy5\left( C19:Slide 2, technial repliate 1 \right)})$$

$$M_{Technical replicate 2}= {Log}_{2}\left( \frac{Cy5\left( VC:Slide 1, technial repliate 2 \right) \times Cy3\left( VC:Slide 2, technial repliate 2 \right)}{Cy3\left( C19:Slide 1, technial repliate 2 \right) \times Cy5\left( C19:Slide 2, technial repliate 2 \right)} \right)$$

Average M

$$Average M=\frac{M_{Technical replicate 1}+M_{Technical replicate 1}}{2}$$

The standard deviation of $M_{Technical replicate 1}$ and $M_{Technical replicate 2}$ was calculated and proteins that had a standard deviation of less that 10% of $Average M$, or has an $Average M$ of greater than ±0.2 were considered statistically differentially expressed and were included in further analyses.

**IX) Protein expression analysis**

GeneVenn was used to find common genes that were affected by the compounds between the MCF-7 and MDA-MB-231 cells [[6](#_ENREF_6)].

**Morphological examination: Fluorescent microscopy**

Exponentially growing MCF-7, MDA-MB-231 and MCF-12A cells were seeded at 350 000 cells per well in 6-well plates. After 24 h attachment the medium was discarded and the cells exposed to 200 nM of ESE-16 and incubated for 24 h or 48 h. For fluorescent microscopy, 0.5 mL of Hoechst 33342 solution (3.5 µg/mL in PBS) was added after time of exposure to the medium to give a final concentration of 0.9 µM and incubated for 30 min at 37 °C in a CO_2_ incubator. After 25 min, 0.5 mL of acridine orange solution **(**4 µg/mL) were added to the medium to give a final concentration of 1 µg/mL and incubated for 5 min at 37°C. After 30 min, the medium was removed and the cells were carefully rinsed three times with PBS before being immersed in clean PBS (1 mL). The cells were examined with a Zeiss inverted Axiovert CFL40 microscope and Zeiss Axiovert MRm monochrome camera under Zeiss Filter 2 for Hoechst 33342 (blue) stained celssand Zeiss Filter 9 for acridine orange-stained cells (green). In order to prevent fluorescent dye quenching, all procedures were performed in a dark room (Carl Zeiss (Pty) Ltd., Johannesburg, South Africa).

**Annexin V expression**

Exponentially growing MCF-12A, MCF-7 and MDA-MB-231 cells were seeded at 350 000 cells per well in 6-well plates. After 24 h attachment the medium was discarded and the cells were exposed to 200 nM of ESE-16 and incubated for the desired period of time. After the allotted time of treatment, cells were trypsinized and 500 000 cells were resuspended in 1 mL of 1x binding buffer (pH7.4), HEPES 100.0 mM, NaCl 1.5 M, KCl 50.0 mM, MgCl_2_ 10.0 mM, CaCl_2_ 18.0 mM) and centrifuged at 300×g for 10 min. The supernatant was removed and the cells were resupended in 100 µL of 1x Binding buffer. 10 µL of Annexin V-FITC was added and the samples were incubated for 15 min in the dark at room temperature. After 15 min the cells were washed by adding 1 mL of 1x binding buffer and centrifuged at 300×g for 10 min. The supernatant was carefully removed and the cells were resuspended in 500 µL of 1x binding buffer solution. Annexin V fluorescence (apoptotic cells) was measured with a FACS FC500 System flow cytometer (Beckman Coulter South Africa (Pty) Ltd) equipped with an air-cooled argon laser excited at 488 nm. Experiments were conducted in triplicate and data from at least 10 000 cells were analyzed with Cyflogic (CyFlo Ltd, Turku, Finland) and with WEASEL version 3.0 software (F. Battye, Walter and Eliza Hall Institute (WEHI), Melbourne, Australia).

**Mitochondrial membrane potential**

Exponentially growing MCF-12A, MCF-7 and MDA-MB-231 cells were seeded at 350 000 cells per well in 6-well plates. After 24 h attachment the medium was discarded and the cells were exposed 200 nM of ESE-16 and incubated for the desired period of time. After the allotted time of treatment, cells were trypsinized and 500 000 cells were incubated with 0.5 mL of the MitoCapture™ reagent for 15 min. Fluorescence of the dye was measured with a FACS FC500 measured with a fluorescence activated cell sorting (FACS) FC500 System flow cytometer (Beckman Coulter SA (Pty) Ltd.). Data from at least 10 000 cells were analyzed with Cyflogic (CyFlo Ltd, Turku, Finland) and with WEASEL version 3.0 software (F. Battye, Walter and Eliza Hall Institute (WEHI), Melbourne, Australia).

**Lysosomal stability**

Acridine orange was used to measure the functional state of lysosomes. AO is lysosomotropic and emits red light at high concentrations (when present in functional lysosomes) and green light when present in the cytosol [[22](#_ENREF_22),[23](#_ENREF_23)].

Exponentially growing MCF-12A, MCF-7 and MDA-MB-231 cells was seeded at 750 000 cells per 25cm^2^ flask. After 24 h attachment the medium was discarded and the cells exposed to 200 nM of ESE-16 and incubated for 24 h. After the allotted time of treatment, cells were trypsinized and 500 000 cells were incubated with 5 µg/mL of AO for 15 min at 37°C. Fluorescence of the dye was measured with a FACS FC500 measured with a fluorescence activated cell sorting (FACS) FC500 System flow cytometer (Beckman Coulter SA (Pty) Ltd.). Data from at least 10 000 cells were analyzed with Cyflogic (CyFlo Ltd, Turku, Finland) and with WEASEL version 3.0 software (F. Battye, Walter and Eliza Hall Institute (WEHI), Melbourne, Australia).

**Active caspase 3 and caspase 7 expression**

Exponentially growing MCF-12A, MCF-7 and MDA-MB-231 cells were seeded at 1.5x10^6^ cells per 25cm^2^ flask. After 24 h attachment the medium was discarded and the cells exposed to 200 nM of ESE-16 and incubated for 24 h. After the allotted time of treatment, cells were trypsinized and 500 000 cells were centrifuged to discard the media. The cells were suspended in wash buffer and centrifuged again. The supernatant was removed and the cells were suspended in Fixation buffer (0.1% formaldehyde) and incubated for 20 mins at room temperature. Afterwards the cells were centrifuged and the supernatant was removed. The cells were suspended in Assay buffer (1% Bovine Serum Albumin) and centrifuged. Ice cold Permeabilization buffer (500 µL 100% methanol) was added and the cells were suspended and incubated on ice for 10 min. Afterwards the cells were spun down and washed by suspending the cells in Assay buffer. The cells were spun down and suspended in 100 µL of Assay buffer with a 1:100 dilution of the primary antibody, rabbit anti-active caspase 3 or with 15 µg/mL of the primary antibody, rabbit anti-active caspase 7. The solution was incubated for 90 min on ice, after which 900 µL of Assay buffer was added to wash the cells. The cells were centrifuged and washed twice with 500 µL Assay buffer. The cells were spun down and then suspended in 100 µL of Assay buffer and 0.2 µg/mL of the an anti-rabbit antibody conjugated with the Dylight™ 488 fluorochrome. The samples were incubated for 1 hr in the dark on ice. Afterwards the 900 µL of Assay buffer was added to wash the cells. The cells were centrifuged and washed twice with 500 µL Assay buffer. The cells were spun down and then suspended in 500 µL of Assay buffer. Fluorescence of the FL1 channel was measured with a fluorescence activated cell sorting (FACS) FC500 measured with a fluorescence activated cell sorting (FACS) FC500 System flow cytometer (Beckman Coulter SA (Pty) Ltd.). Data from at least 10 000 cells were analyzed with Cyflogic (CyFlo Ltd, Turku, Finland).

***Phosphorylation of Bcl-2 at Serine 70***

Exponentially growing MCF-12A, MCF-7 and MDA-MB-231 cells were seeded at 1.5x10^6^ cells per 25cm^2^ flask. After 24 h attachment the medium was be discarded and the cells exposed to 200 nM of ESE-16 and incubated for 24 h. After the allotted time of treatment, cells were trypsinized and 500 000 cells were centrifuged to discard the media. The cells were suspended in wash buffer and centrifuged again. The supernatant was removed and the cells were suspended in fixation buffer (0.1% formaldehyde) and incubated for 20 mins at room temperature. Afterwards the cells were centrifuged and the supernatant was removed. The cells were suspended in Assay buffer (1% Bovine Serum Albumin) and centrifuged. Ice cold permeabilization buffer (500 µL 100% methanol) was added and the cells were suspended and incubated on ice for 10 mins. Afterwards the cells were spun down and washed by suspending the cells in Assy buffer. The cells were spun down and suspended in 100 µL of Assay buffer with an anti-Bcl-2 antibody conjugated to AlexaFluor® 488 and an anti pBcl-2 (ser70) conjugated to Phycoerythrin. The solution was incubated for 60 mins on ice in the dark, after which 900 µL of Assay buffer was added to wash the cells. The cells were centrifuged and washed twice with 500 µL Assay buffer. Afterwards the 900 µL of Assay buffer was added to wash the cells. The cells were centrifuged and washed twice with 500 µL Assay buffer. The cells were spun down and then suspended in 500 µL of Assay buffer. Fluorescence of the FL1 (for measuring the Bcl-2 antibody) and FL3 (for measuring the pBcl-2, Ser 70) channel were measured with a fluorescence activated cell sorting (FACS) FC500 measured with a fluorescence activated cell sorting (FACS) FC500 System flow cytometer (Beckman Coulter SA (Pty) Ltd.). Data from at least 10 000 cells were analyzed with Cyflogic (CyFlo Ltd, Turku, Finland).

References

1. Wettenhall JM, Smyth GK (2004) limmaGUI: a graphical user interface for linear modeling of microarray data. Bioinformatics 20: 3705.

2. Smyth G (2005) Limma: linear models for microarray data. Bioinformatics and computational biology solutions using R and bioconductor: 397-420.

3. Smyth GK, Speed T (2003) Normalization of cDNA microarray data. Methods 31: 265-273.

4. Benjamini Y, Hochberg Y (1995) Controlling the false discovery rate: a practical and powerful approach to multiple testing. Journal of the Royal Statistical Society Series B (Methodological): 289-300.

5. Nogales-Cadenas R, Carmona-Saez P, Vazquez M, Vicente C, Yang X, et al. (2009) GeneCodis: interpreting gene lists through enrichment analysis and integration of diverse biological information. Nucleic acids research 37: W317.

6. Pirooznia M, Nagarajan V, Deng Y (2007) GeneVenn—a web application for comparing gene lists using Venn diagrams. Bioinformation 1: 420-422.

7. Edwards D (2003) Non-linear normalization and background correction in one-channel cDNA microarray studies. Bioinformatics 19: 825-833.

8. Moschos SJ, Smith AP, Mandic M, Athanassiou C, Watson-Hurst K, et al. (2007) SAGE and antibody array analysis of melanoma-infiltrated lymph nodes: identification of Ubc9 as an important molecule in advanced-stage melanomas. Oncogene 26: 4216-4225.
