# Supplementary material for: Signaling Pathways of ESE-16, an Antimitotic and Anticarbonic Anhydrase Estradiol Analog, in Breast Cancer Cells
Source: PLoS One. 2013 Jan 31;8(1):e53853. doi: 10.1371/journal.pone.0053853 (PMC3561402; doi:10.1371/journal.pone.0053853)
Supplement: Supporting Information S2 — Extensive list of differentially expressed genes (adjusted P- value <0.05) mapped to functional cellular pathways in MCF-7, MDA-MB-231 and MCF12A cells exposed to ESE-15-one (140 nM), ESE-15-ol (50 nM), and ESE-16 (200 nM) for 24 h. (DOCX) [file pone.0053853.s002.docx]

| RNA | name |  |  |  |  |
| --- | --- | --- | --- | --- | --- |
|  |  |  | ESE-16 | ESE-16 | ESE-16 |
|  |  |  | MCF-7 | MDA-MB-231 | MCF-12A |
|  |  |  | 200 nM | 200 nM | 200 nM |
| Cell growth, apoptosis, autophagy | |  |  |  |  |
| NM_014417 | BBC3/PUMA | BCL2 binding component 3 | 1.66 | 1.7 | 1.81 |
| NM_001731 | BTG1 | B-cell translocation gene 1 anti-proliferative | 1.32 | 0.78 | 0.45 |
| NM_004417 | DUSP1 | Dual specificity phosphatase 1 | 1.97 | 2.05 | 1.33 |
| NM_033285 | TP53INP1 | Tumor protein p53 inducible nuclear protein 1 | 1.18 | 0.74 | 0.99 |
| NM_145008 | YPEL4 | Yippee-like 4 | 1.5 | 2.27 | 1.12 |
| AF100640 | MB2 | Metastasis related protein | 0.98 | 3.68 | 1.65 |
| NM_006533 | MIA | Melanoma inhibitory activity | 0.43 | 0.66 | 0.62 |
| NM_201444 | DGKA | Diacylglycerol kinase alpha 80kda | 0.67 | 0.52 | 0.44 |
| NM_001002914 | KCTD11 | Potassium channel tetramerisation domain containing 11 | 0.64 | 0.7 | 0.4 |
|  |  |  |  |  |  |
| Cell cycle and DNA repair | |  |  |  |  |
| NM_001007793 | BUB3 | Budding uninhibited by benzimidazoles 3 homolog | -1.32 | -0.78 | -0.45 |
| NM_001786 | CDC2 | Cell division cycle 2 G1 to S and G2 to M | -0.91 | -1.24 | -0.84 |
| NM_030928 | CDT1 | Chromatin licensing and DNA replication factor 1 | -0.52 | -0.96 | -0.56 |
| NM_005573 | LMNB1 | Lamin B1 | -0.98 | -1.64 | -1.02 |
| NM_006739 | MCM5 | MCM5 minichromosome maintenance deficient 5 cell division cycle 46 | -0.57 | -0.89 | -0.55 |
| NM_004153 | ORC1L | Origin recognition complex subunit 1-like | -0.86 | -1.23 | -0.99 |
| NM_002592 | PCNA | Proliferating cell nuclear antigen | -0.51 | -1.1 | -0.51 |
| NM_006622 | PLK2 | Polo-like kinase 2 | 0.96 | 1.23 | 0.99 |
| NM_020675 | SPBC25 | Spindle pole body component 25 homolog | -0.75 | -0.69 | -0.55 |
| NM_006879 | MDM2 | Mdm2 transformed 3T3 cell double minute | 1.23 | 1.85 | 1.13 |
| CLTCL1 | RIIAD1 | Clathrin, heavy chain-like 1 | 0.63 | 0.86 | 0.61 |
|  |  |  |  |  |  |
| Oxidative stress related | |  |  |  |  |
| NM_002133 | HMOX1 | Heme oxygenase 1 | 0.77 | 1.15 | 0.95 |
| NM_002970 | SAT1/SSAT | Spermidine/spermine N1-acetyltransferase 1 | 0.8 | 0.81 | 0.68 |
|  |  |  |  |  |  |
| Epigenetics-related and chromatin modifications | | |  |  |  |
| NM_001379 | DNMT1 | DNA methyl transferase 1 | -0.45 | -0.58 | -0.64 |
| NM_003642 | HAT1 | Histone acetyltransferase 1 | -0.4 | -1.12 | -0.36 |
| BQ683489 | HIST1H2BD | Histone cluster 1 h2bd | -1.29 | -1.98 | -0.59 |
| NM_003537 | HIST1H3B | Histone cluster 1 H3b | -1.19 | -1.56 | -0.97 |
| NM_003534 | HIST1H3G | Histone cluster 1 H3g | -0.67 | -1.61 | -0.48 |
| NM_175065 | HIST2H2AB | Histone cluster 2 h2ab | -0.37 | -1.02 | -0.45 |
| NM_003517 | HIST2H2AC | Histone cluster 2 h2ac | -1.62 | -1.68 | -0.94 |
| NM_001025303 | HIST2H3PS2 | Histone cluster 2 H3 pseudogene 2 | -0.95 | -1.78 | -0.59 |
| DB010344 | HIST3H2BB | Histone cluster 3 h2bb | -1.57 | -2.16 | -0.75 |
|  |  |  |  |  |  |
| Structural |  |  |  |  |  |
| XR_018167 | LOC647690 | Tubulin, alpha 1 ( | -1.1 | -0.61 | -1.03 |
| NM_006306 | SMC1A | Structural maintenance of chromosomes 1A | -0.66 | -0.65 | -0.6 |
| NM_079836 | TUBA2 | Tubulin, alpha 2 (TUBA2), transcript variant 2, | -1.11 | -0.56 | -1.11 |
| NM_006088 | TUBB2C | Tubulin beta 2C | -1.32 | -0.8 | -1.09 |
| ENST00000355609 | IFFO2 | Intermediate filament family orphan 2 | 0.94 | 0.93 | 0.57 |
|  |  |  |  |  |  |
| Regulation of Transcription | |  |  |  |  |
| NM_002128 | HMGB1 | High-mobility group box 1 | -0.67 | -0.7 | -0.75 |
| NM_000600 | IL6 | Interleukin 6 | 1.68 | 3.66 | 1.93 |
| NM_016269 | LEF1 | Lymphoid enhancer-binding factor 1 | 0.66 | 0.93 | 0.54 |
| NM_003168 | SUPT4H1 | Suppressor of Ty 4 homolog 1 | 0.81 | 0.94 | 0.83 |
| NM_003246 | THBS1 | Thrombospondin 1 | 0.47 | 1.14 | 0.5 |
| X66087 | MYBL1 | V-Myb myeloblastosis viral oncogene homolog (avian)-like 1 | -0.7 | -1.03 | -0.94 |
| NM_172164 | NASP | Nuclear autoantigenic sperm protein | -0.46 | -0.64 | -0.44 |
| NM_021005 | NR2F2 | Nuclear receptor subfamily 2 group F member 2 | -0.91 | -1.33 | -0.77 |
| NM_006191 | PA2G4 | Proliferation-associated 2G4 38kda | -0.53 | -0.6 | -0.54 |
| NM_153695 | ZNF367 | Zinc finger protein 367 | -0.83 | -1.12 | -0.72 |
| BC011243 | ZNF587 | Zinc finger protein 587 | -0.62 | -0.84 | -0.53 |
| NM_006509 | RELB | v-Rel reticuloendotheliosis viral oncogene homolog B | 0.77 | 1.06 | 0.74 |
|  |  |  |  |  |  |
| Metabolism |  |  |  |  |  |
| AF132203 | AF132203 | Stearoyl-coa desaturase (delta-9-desaturase) | -0.74 | -2.22 | -0.87 |
| NR_003262 | FDPSL2A | Farnesyl diphosphate synthase-like 2 | -0.49 | -1.14 | -0.67 |
| XR_018717 | FDPSL4 | Farnesyl diphosphate synthetase-like 4 | -0.49 | -1.38 | -0.74 |
| NM_001386 | DPYSL2 | Dihydropyrimidinase-like 2 | -0.92 | -0.7 | -1.25 |
| NM_017712 | PGPEP1 | Pyroglutamyl-peptidase I | 0.49 | 0.83 | 0.54 |
| NM_006623 | PHGDH | Phosphoglycerate dehydrogenase | -0.7 | -0.84 | -0.51 |
| NM_014298 | QPRT | Quinolinate phosphoribosyltransferase | -0.54 | -1.25 | -0.49 |
| NM_001034 | RRM2 | Ribonucleotide reductase M2 polypeptide | -0.52 | -0.94 | -0.58 |
| NM_001025390 | AMPD3 | Adenosine monophosphate deaminase, transcript variant 3 | 1.35 | 1.51 | 0.44 |
| NM_019885 | CYP26B1 | Cytochrome P450, family 26, subfamily B, polypeptide 1 | 1.06 | 1.58 | 1.78 |
| NM_002130 | HMGCS1 | 3-Hydroxy-3-methylglutaryl-Coenzyme A synthase 1 | -0.97 | -1.95 | -0.96 |
|  |  |  |  |  |  |
| Transmembrane |  |  |  |  |  |
| NM_153342 | TMEM150 | Transmembrane protein 150 | 0.59 | 1.32 | 0.98 |
| NM_001040455 | SIDT2 | SID1 transmembrane family, member 2 | 0.8 | 1.4 | 0.42 |
| NM_005780 | LHFP | Lipoma HMGIC fusion partner | 0.59 | 1.2 | 0.64 |
|  |  |  |  |  |  |
| Extracellular matrix | |  |  |  |  |
| NM_003255 | TIMP2 | TIMP metallopeptidase inhibitor 2 | 1.38 | 1.18 | 0.4 |
|  |  |  |  |  |  |
| Other |  |  |  |  |  |
| AB014766 | DERP12 | Dermal papilla derived protein 12 | 1.37 | 3.76 | 1.42 |
| AK055372 | LINC00461 | Long intergenic non-protein coding RNA 461 | -0.53 | -0.97 | -0.41 |
| NM_147129 | ALS2CL | ALS2 C-terminal like | 0.54 | 0.37 | 0.82 |
| XR_019062 | LOC643513 | Importin alpha-2 subunit | -0.87 | -1.02 | -0.77 |
| NM_022978 | SERF1B | Small EDRK-rich factor 1B (centromeric) | 0.64 | 0.95 | 0.65 |
| NM_013282 | UHRF1 | Ubiquitin-like containing PHD and RING finger domains 1 | -0.99 | -1.59 | -1.06 |
|  |  |  |  |  |  |
|  |  |  |  |  |  |
|  |  |  |  |  |  |
| Refseq | Gene | Description | log M (Diff expressed) | | |
| RNA | name |  |  |  |  |
|  |  |  | ESE-16 | ESE-16 | ESE-16 |
|  |  |  | MCF-7 | MDA-MB-231 | MCF-12A |
|  |  |  | 200 nM | 200 nM | 200 nM |
| Cell growth, apoptosis, autophagy | |  |  |  |  |
| AF100640 | AF100640 | Metastasis related protein | 0.98 |  | 1.65 |
| NM_147129 | ALS2CL | ALS2 C-terminal like | 0.54 | 0.82 | 0.37 |
| NM_138764 | BAX | BCL2-associated X protein transcript variant epsilon |  |  | 0.49 |
| NM_014417 | BBC3/PUMA | BCL2 binding component 3 | 1.66 | 1.7 | 1.81 |
| NM_138621 | BCL2L11 | BCL2-like 11 |  | 0.61 |  |
| NM_015367 | BCL2L13 | BCL2-like 13 nuclear gene encoding mitochondrial protein |  |  | 0.42 |
| NM_020993 | BCL7A | B-cell CLL/lymphoma 7A transcript variant 1 |  |  | -0.41 |
| NM_007295 | BRCA1 | Breast cancer 1 | -0.5 |  |  |
| NM_001018055 | BRCC3 | BRCA1/BRCA2-containing complex subunit 3 transcript variant 2 | -0.5 |  | -0.41 |
| NM_001731 | BTG1 | B-cell translocation gene 1 anti-proliferative | 1.32 | 0.78 | 0.45 |
| NM_170589 | CASC5 | Cancer susceptibility candidate 5 |  |  | -0.52 |
| NM_001814 | CTSC | Cathepsin C |  |  |  |
| NM_004083 | DDIT3 | DNA-damage-inducible transcript 3 | 0.5 | 1.44 | 0.58 |
| NM_001343 | DAB2 | Disabled homolog 2 mitogen-responsive phosphoprotein | 0.78 |  | 0.59 |
| NM_004093 | EFNB2 | Ephrin-B2 |  | 0.87 |  |
| NM_004438 | EPHA4 | EPH receptor A4 | 0.87 | 0.81 | 0.46 |
| NM_004440 | EPHA7 | EPH receptor A7 | -0.51 | -0.79 | -0.48 |
| NM_001924 | GADD45A | Growth arrest and DNA-damage-inducible alpha | 0.41 | 0.66 |  |
| NM_201612 | IKIP | IKK interacting protein transcript variant 2 | 0.68 | 0.61 | 0.41 |
| NR_002819 | MALAT1 | Metastasis associated lung adenocarcinoma transcript 1 | 0.65 |  |  |
| NM_006879 | MDM2 | Mdm2 transformed 3T3 cell double minute 2 | 1.23 | 1.85 | 1.13 |
| NM_006533 | MIA | Melanoma inhibitory activity | 0.43 | 0.66 | 0.62 |
| CR613982 | MIA3 | Melanoma inhibitory activity protein 3 | 0.43 |  |  |
| NM_004739 | MTA2 | Metastasis associated 1 family member 2 |  |  | -0.52 |
| NM_001077493 | NFKB2 | Nuclear factor of kappa light polypeptide gene enhancer in B-cells 2 |  | 0.55 |  |
| NM_020529 | NFKBIA/IκBα | Nuclear factor of kappa light polypeptide gene enhancer in B-cells inhibitor alpha | 0.96 | 1.27 | 0.74 |
| NM_001031689 | PLAA | Phospholipase A2-activating protein |  |  | -0.4 |
| NM_000314 | PTEN | Phosphatase and tensin homolog |  | 0.72 |  |
| NM_006509 | RELB | v-Rel reticuloendotheliosis viral oncogene homolog B | 0.77 | 1.06 | 0.74 |
| NM_152756 | RICTOR | Rapamycin-insensitive companion of mTOR |  | -1.04 |  |
| NM_003942 | RPS6KA4 | Ribosomal protein S6 kinase 90kDa polypeptide 4 | -0.38 | -0.39 | -0.64 |
| U46752 | SQSTM1 | Human phosphotyrosine independent ligand | 0.98 | 0.52 |  |
| ENST00000366930 | TGFB2 | Transforming growth factor beta-2 precursor TGF-beta-2 |  |  | 0.42 |
| NM_001024847 | TGFBR2 | Transforming growth factor beta receptor II | 0.87 |  | 0.56 |
| NM_014452 | TNFRSF21 | Tumor necrosis factor receptor superfamily member 21 | 0.5 |  |  |
| NM_033285 | TP53INP1 | Tumor protein p53 inducible nuclear protein 1 | 1.18 | 0.74 | 0.99 |
| NM_016058 | TPRKB | TP53RK binding protein |  | -0.54 |  |
| NM_003295 | TPT1 | Tumor protein translationally-controlled 1 | 0.42 |  | 0.4 |
| NM_021643 | TRIB2 | Tribbles homolog 2 |  | 0.79 |  |
| NM_000548 | TSC2 | Tuberous sclerosis 2 | 0.45 | 0.6 |  |
| NM_001025366 | VEGFA | Vascular endothelial growth factor Atranscript variant 1 | 0.35 |  | 0.38 |
| NM_001025370 | VEGFA | Vascular endothelial growth factor A transcript variant 6 | -0.38 |  |  |
| NM_003377 | VEGFB | Vascular endothelial growth factor B |  |  | -0.47 |
| NM_031477 | YPEL3 | Yippee-like 3 | 0.61 |  |  |
| NM_145008 | YPEL4 | Yippee-like 4 | 1.5 | 2.27 | 1.12 |
|  |  |  |  |  |  |
| Cell cycle and DNA repair | |  |  |  |  |
| NM_004336 | BUB1 | BUB1 budding uninhibited by benzimidazoles 1 homolog |  |  | -0.75 |
| NM_001211 | BUB1B | BUB1 budding uninhibited by benzimidazoles 1 homolog beta |  | -0.53 |  |
| NM_001237 | CCNA2 | Cyclin A2 |  | -0.69 |  |
| NM_031966 | CCNB1 | Cyclin B1 |  |  |  |
| NM_004701 | CCNB2 | Cyclin B2 |  | -0.66 |  |
| NM_053056 | CCND1 | Cyclin D1 | -0.38 |  |  |
| NM_003858 | CCNK | Cyclin K | -0.47 | -1.02 |  |
| NM_020307 | CCNL1 | Cyclin L1 |  |  | 0.39 |
| NM_020739 | CCPG1 | Cell cycle progression 1 |  |  | -0.54 |
| NM_001786 | CDC2 | Cell division cycle 2 G1 to S and G2 to M | -0.91 | -1.24 | -0.84 |
| NM_001255 | CDC20 | Cell division cycle 20 homolog |  | -0.5 |  |
| NM_033487 | CDC2L1 | Cell division cycle 2-like 1 | -0.69 | -0.82 |  |
| NM_003718 | CDC2L5 | Cell division cycle 2-like 5 |  | -0.54 |  |
| NM_044472 | CDC42 | Cell division cycle 42 transcript variant 2 |  |  | 0.39 |
| NM_005851 | CDK2AP2 | CDK2-associated protein 2 |  |  | -0.43 |
| NM_001261 | CDK9 | Cyclin-dependent kinase 9 |  | 0.83 |  |
| NM_001800 | CDKN2D | Cyclin-dependent kinase inhibitor 2D |  |  |  |
| NM_030928 | CDT1 | Chromatin licensing and DNA replication factor 1 | -0.52 | -0.96 | -0.56 |
| NM_001810 | CENPB | Centromere protein B 80kda |  |  | -0.52 |
| NM_001813 | CENPE | Centromere protein E 312kda |  |  |  |
| NM_018451 | CENPJ | Centromere protein J | -0.5 |  |  |
| NM_033319 | CENPL | Centromere protein L |  |  | -0.36 |
| NM_018455 | CENPN | Centromere protein N |  |  |  |
| NM_025082 | CENPT | Centromere protein T |  |  |  |
| NM_018131 | CEP55 | Centrosomal protein 55kda |  |  |  |
| NM_014679 | CEP57 | Centrosomal protein 57kda |  | -0.62 |  |
| NM_004365 | CETN3 | Centrin EF-hand protein 3 |  | -0.69 |  |
| NM_001826 | CKS1B | CDC28 protein kinase regulatory subunit 1B |  | -0.63 |  |
| NM_199077 | CNNM2 | Cyclin M2 | -1.31 |  |  |
| NM_020184 | CNNM4 | Cyclin M4 |  |  |  |
| BC111740 | DNA2L | DNA2 DNA replication helicase 2-like |  |  |  |
| NM_004947 | DOCK3 | Dedicator of cytokinesis 3 | 0.49 |  |  |
| NM_014705 | DOCK4 | Dedicator of cytokinesis 4 |  |  |  |
| NM_012291 | ESPL1 | Extra spindle pole bodies homolog 1 |  | -0.57 |  |
| NM_018063 | HELLS | Helicase lymphoid-specific |  |  | -0.52 |
| NM_005733 | KIF20A | Kinesin family member 20A |  | -0.5 | -0.67 |
| NM_007317 | KIF22 | Kinesin family member 22 |  |  |  |
| NM_017576 | KIF27 | Kinesin family member 27 |  |  | -0.78 |
| NM_007054 | KIF3A | Kinesin family member 3A | -0.46 |  |  |
| NM_004798 | KIF3B | Kinesin family member 3B | 0.4 |  |  |
| NM_012310 | KIF4A | Kinesin family member 4A |  | -0.51 |  |
| NM_002263 | KIFC1 | Kinesin family member C1 |  | -0.75 |  |
| NM_006101 | KNTC2 | Kinetochore associated 2 |  |  | -0.43 |
| NM_005572 | LMNA | Lamin A/C transcript variant 2 |  |  | -0.48 |
| NM_005573 | LMNB1 | Lamin B1 | -0.98 | -1.64 | -1.02 |
| NM_032737 | LMNB2 | Lamin B2 |  |  |  |
| NM_002358 | MAD2L1 | MAD2 mitotic arrest deficient-like 1 |  | -0.59 | -0.48 |
| NM_006341 | MAD2L2 | MAD2 mitotic arrest deficient-like 2 | -0.38 |  | -0.41 |
| NM_006739 | MCM5 | MCM5 minichromosome maintenance deficient 5 cell division cycle 46 | -0.57 | -0.89 | -0.55 |
| NM_198175 | NME1 | Non-metastatic cells 1 protein transcript variant 1 | 0.37 |  | 0.37 |
| NM_145697 | NUF2 | NUF2 NDC80 kinetochore complex component homolog |  | -0.84 |  |
| NM_016359 | NUSAP1 | Nucleolar and spindle associated protein 1 | -0.53 |  | -0.71 |
| NM_004153 | ORC1L | Origin recognition complex subunit 1-like | -0.86 | -1.23 | -0.99 |
| NM_002553 | ORC5L | Origin recognition complex subunit 5-like |  |  | -0.4 |
| NM_002577 | PAK2 | p21 -activated kinase 2 | -0.51 | -0.78 |  |
| NM_002592 | PCNA | Proliferating cell nuclear antigen | -0.51 | -1.1 | -0.51 |
| NM_002648 | PIM1 | Pim-1 oncogene |  |  | 0.87 |
| NM_005030 | PLK1 | Polo-like kinase 1 |  |  |  |
| NM_006622 | PLK2 | Polo-like kinase 2 | 0.96 | 1.23 | 0.99 |
| NM_014264 | PLK4 | Polo-like kinase 4 |  |  |  |
| NM_005053 | RAD23A | RAD23 homolog A |  |  | -0.47 |
| NM_002874 | RAD23B | RAD23 homolog B | -0.51 | -0.77 |  |
| NM_005732 | RAD50 | RAD50 homolog | -0.5 |  | -0.58 |
| NM_012415 | RAD54B | RAD54 homolog B |  |  | 1.64 |
| NM_003579 | RAD54L | RAD54-like |  |  |  |
| NM_001034836 | RDM1 | RAD52 motif 1 |  | -0.66 |  |
| NM_004260 | RECQL4 | RECQ protein-like 4 | -0.42 | -0.59 |  |
| NM_002913 | RFC1 | Replication factor C 1 | -1.1 |  | -0.86 |
| NM_181471 | RFC2 | Replication factor C 2 |  | -0.54 | -0.59 |
| NM_002916 | RFC4 | Replication factor C 4 |  | -0.62 |  |
| NM_006306 | SMC1A | Structural maintenance of chromosomes 1A | -0.66 | -0.65 | -0.6 |
| NM_001042550 | SMC2 | Structural maintenance of chromosomes 2 |  | -0.83 | -0.51 |
| NM_005445 | SMC3 | Structural maintenance of chromosomes 3 | -0.38 |  | -0.57 |
| NM_024624 | SMC6 | Structural maintenance of chromosomes 6 |  |  | -0.47 |
| NM_020675 | SPBC25 | Spindle pole body component 25 homolog | -0.75 | -0.69 | -0.55 |
| NM_203401 | STMN1 | Stathmin 1/oncoprotein 18 | -0.33 | -0.8 |  |
| NM_030795 | STMN4 | Stathmin-like 4 |  |  | 0.98 |
| NM_003286 | TOP1 | Topoisomerase 1 |  |  | -0.41 |
| NM_007027 | TOPBP1 | Topoisomerase |  |  | -0.61 |
|  |  |  |  |  |  |
| Oxidative stress related | |  |  |  |  |
| NM_002032 | FTH1 | Ferritin heavy polypeptide 1 | 0.53 | 0.65 | 0.42 |
| NM_002083 | GPX2 | Glutathione peroxidase 2 | -0.49 |  | -0.62 |
| NM_000846 | GSTA2 | Glutathione S-transferase A2 |  |  | 0.59 |
| NM_014685 | HERPUD1 | Homocysteine-inducible endoplasmic reticulum |  | 1.12 |  |
|  |  | stress-inducible ubiquitin-like domain 1 |  |  |  |
| NM_002133 | HMOX1 | Heme oxygenase 1 | 0.77 | 1.15 | 0.95 |
| NM_145791 | MGST1 | Microsomal glutathione S-transferase 1 transcript variant 1c |  | -0.9 |  |
| NM_000625 | NOS2A | Nitric oxide synthase 2A |  | 0.75 | 0.36 |
| NM_004337 | OSGIN2 | Oxidative stress induced growth inhibitor family member 2 |  | -0.5 |  |
| NM_005109 | OXSR1 | Oxidative-stress responsive 1 | -0.47 |  | -0.57 |
| NM_002574 | PRDX1 | Peroxiredoxin 1 transcript variant 1 |  |  | -0.43 |
| NM_005809 | PRDX2 | Peroxiredoxin 2 nuclear gene encoding mitochondrial protein | 0.44 |  |  |
| NM_006793 | PRDX3 | Peroxiredoxin 3 |  | -1.06 |  |
| NM_002970 | SAT1/SSAT | Spermidine/spermine N1-acetyltransferase 1 | 0.8 | 0.81 | 0.68 |
| NM_175839 | SMOX | Spermine oxidase |  | 0.91 |  |
| NM_001024465 | SOD2 | Superoxide dismutase 2 |  | 1.1 |  |
| NM_006819 | STIP1 | Stress-induced-phosphoprotein 1 | -0.58 |  | -0.47 |
| NM_005783 | TXNDC9 | Thioredoxin domain containing 9 | -0.39 |  |  |
| NM_004786 | TXNL1 | Thioredoxin-like 1 | -0.37 |  |  |
|  |  |  |  |  |  |
| Phosphatases |  |  |  |  |  |
| NM_001008392 | CTDSPL | Carboxy-terminal domain small phosphatase-like protein |  |  | -0.77 |
| NM_004417 | DUSP1 | Dual specificity phosphatase 1 | 1.97 | 2.05 | 1.33 |
| NM_001007271 | DUSP13 | Dual specificity phosphatase 13 |  | 0.76 |  |
| NM_080611 | DUSP15 | Dual specificity phosphatase 15 |  | 0.86 |  |
| NM_004418 | DUSP2 | Dual specificity phosphatase 2 |  | 1.62 |  |
| NM_003713 | PPAP2B | Phosphatidic acid phosphatase type 2B |  | 1.43 |  |
| NM_177983 | PPM1G | Protein phosphatase 1G magnesium-dependent gamma isoform | -0.51 |  |  |
| NM_002710 | PPP1CC | Protein phosphatase 1 catalytic subunit gamma isoform | -0.47 |  |  |
| NM_032105 | PPP1R12B | Protein phosphatase 1 regulatory subunit 12B | 0.82 | 0.81 |  |
| NM_033256 | PPP1R14A | Protein phosphatase 1 regulatory subunit 14A | 0.53 |  |  |
| NM_138689 | PPP1R14B | Protein phosphatase 1 regulatory subunit 14B | -0.53 |  |  |
| NM_002720 | PPP4C | Protein phosphatase 4 catalytic subunit |  | 0.62 | -0.44 |
| NM_174907 | PPP4R2 | Protein phosphatase 4 regulatory subunit 2 | -0.45 |  | -0.44 |
| NM_000314 | PTEN | Phosphatase and tensin homolog |  | 0.74 |  |
| NM_003463 | PTP4A1 | Protein tyrosine phosphatase type IVA | 0.69 | 0.7 | 0.45 |
| NM_080391 | PTP4A2 | Protein tyrosine phosphatase type IVA member 2 |  |  | -0.64 |
| NM_016395 | PTPLAD1 | Protein tyrosine phosphatase-like A domain containing 1 | -0.39 |  |  |
| NM_080685 | PTPN13 | Protein tyrosine phosphatase non-receptor type 13 |  | 0.69 |  |
| NM_005401 | PTPN14 | Protein tyrosine phosphatase non-receptor type 14 | -0.92 |  | -0.82 |
| NM_002829 | PTPN3 | Protein tyrosine phosphatase non-receptor type 3 | 0.45 |  |  |
| NM_002830 | PTPN4 | Protein tyrosine phosphatase non-receptor type 4 |  |  | -0.42 |
| NM_006504 | PTPRE | Protein tyrosine phosphatase receptor type E | 0.56 |  | -0.5 |
| NM_002840 | PTPRF | Protein tyrosine phosphatase receptor type F |  | 0.94 | 0.63 |
| NM_002843 | PTPRJ | Protein tyrosine phosphatase receptor type J |  | 0.9 |  |
| NM_002844 | PTPRK | Protein tyrosine phosphatase receptor type K |  | 1.3 |  |
| NM_002845 | PTPRM | Protein tyrosine phosphatase receptor type M | 2.35 |  |  |
| NM_030791 | SGPP1 | Sphingosine-1-phosphate phosphatase 1 |  | -0.92 | -0.51 |
|  |  |  |  |  |  |
| Kinases |  |  |  |  |  |
| NM_152221 | CSNK1E | Casein kinase 1 epsilon | -0.97 |  | -0.63 |
| NM_201444 | DGKA | Diacylglycerol kinase alpha 80kda | 0.67 | 0.52 | 0.44 |
| NM_003646 | DGKZ | Diacylglycerol kinase zeta 104kda |  | -0.49 | -0.45 |
| NM_001004023 | DYRK3 | dual-specificity tyrosine--phosphorylation regulated kinase 3 |  | 0.79 | 0.43 |
| NM_002031 | FRK | Fyn-related kinase | 0.72 | 1.57 |  |
| NM_001005910 | IHPK2 | Inositol hexaphosphate kinase 2 | 0.35 |  |  |
| NM_002227 | JAK1 | Janus kinase 1 | -0.97 |  | -1.05 |
| NM_145109 | MAP2K3 | Mitogen-activated protein kinase kinase 3 |  | 0.57 |  |
| NM_002758 | MAP2K6 | Mitogen-activated protein kinase kinase 6 |  |  | -0.64 |
| ENST00000264777 | MAP3K1 | Mitogen-activated protein kinase kinase kinase 1 |  |  | -0.44 |
| NM_004721 | MAP3K13 | Mitogen-activated protein kinase kinase kinase 13 |  |  | -0.49 |
| NM_004672 | MAP3K6 | Mitogen-activated protein kinase kinase kinase 6 |  |  | -0.45 |
| NM_003618 | MAP4K3 | Mitogen-activated protein kinase kinase kinase kinase 3 |  |  | -0.52 |
| NM_138957 | MAPK1 | Mitogen-activated protein kinase 1 |  |  | -0.44 |
| ENST00000374189 | MAPK8/JNK1 | Mitogen-activated protein kinase 8 |  |  | 0.43 |
| NM_014791 | MELK | Maternal embryonic leucine zipper kinase |  | -1.09 | -0.67 |
| NM_017572 | MKNK2 | MAP kinase interacting serine/threonine kinase 2 |  | 0.79 |  |
| NM_053025 | MYLK | Myosin light chain kinase | -0.64 | -0.8 | -0.66 |
| NM_014840 | NUAK1 | NUAK family SNF1-like kinase 1 | 0.45 |  | 0.49 |
| NM_022731 | NUCKS1 | Nuclear casein kinase and cyclin-dependent kinase substrate 1 | -1.06 | -1.2 | -0.69 |
| NM_152835 | PDIK1L | PDLIM1 interacting kinase 1 like |  | -0.82 |  |
| NM_006823 | PKIA | Protein kinase transcript variant 6 | 0.36 |  | 0.42 |
| NM_182687 | PKMYT1 | Protein kinase membrane associated tyrosine/threonine 1 transcript variant 2 |  |  |  |
| NM_006622 | PLK2 | Polo-like kinase 2 | 0.96 | 1.23 | 0.99 |
| NM_014264 | PLK4 | Polo-like kinase 4 |  |  |  |
| NM_016203 | PRKAG2 | Protein kinase AMP-activated gamma 2 non-catalytic subunit | 0.54 |  |  |
| NM_004157 | PRKAR2A | Protein kinase camp-dependent regulatory type II alpha |  | -0.6 |  |
| NM_002737 | PRKCA | Protein kinase C alpha | 0.36 |  |  |
| NM_012408 | PRKCBP1 | Protein kinase C binding protein 1 transcript variant 2 | -0.47 |  |  |
| NM_145906 | RIOK3 | RIO kinase 3 | 0.6 | 0.69 | 0.4 |
| NM_004586 | RPS6KA3 | Ribosomal protein S6 kinase 90kDa polypeptide 3 | -0.87 | -0.84 | -0.57 |
| NM_005627 | SGK | Serum/glucocorticoid regulated kinase |  | -0.75 | -1.16 |
| NM_000455 | STK11 | Serine/threonine kinase 11 | -0.38 | 1.06 |  |
| NM_001032296 | STK24 | Serine/threonine kinase 24 | 0.5 |  |  |
| NM_015000 | STK38L | Serine/threonine kinase 38 like | 1.03 | 0.64 |  |
| NM_006282 | STK4 | Serine/threonine kinase 4 |  | -0.71 |  |
| NM_032017 | STK40 | Serine/threonine kinase 40 | 0.82 | 1 | 0.56 |
| NM_020791 | TAOK1 | TAO kinase 1 |  | 0.7 |  |
| NM_016281 | TAOK3 | TAO kinase 3 | 0.37 |  |  |
| NM_005781 | TNK2 | Tyrosine kinase non-receptor 2 |  | -0.65 | -0.41 |
| NM_003318 | TTK | TTK protein kinase |  |  |  |
|  |  |  |  |  |  |
| Epigenetics-related and chromatin modifications | | |  |  |  |
| NM_014577 | BRD1 | Bromodomain containing 1 |  | 0.57 |  |
| NM_005104 | BRD2 | Bromodomain containing 2 | -0.41 |  |  |
| NM_176812 | CHMP4B | Chromatin modifying protein 4B |  |  |  |
| NM_014473 | DIMT1L | DIM1 dimethyladenosine transferase 1-like |  |  |  |
| NM_001379 | DNMT1 | DNA methyl transferase 1 | -0.45 | -0.58 | -0.64 |
| NM_013369 | DNMT3L | DNA -methyltransferase 3-like | 0.49 |  | 0.54 |
| NM_032482 | DOT1L | DOT1-like histone H3 methyltransferase | 0.42 |  |  |
| NM_004456 | EZH2 | Enhancer of zeste homolog 2 |  |  |  |
| NM_006026 | H1FX | H1 histone family member X |  | -0.66 | -0.66 |
| NM_012412 | H2AFV | H2A histone family member V |  |  | 0.39 |
| NM_002106 | H2AFZ | H2A histone family member Z | -0.36 | -1.05 |  |
| NM_002107 | H3F3A | H3 histone family 3A | -0.92 |  |  |
| NM_005324 | H3F3B | H3 histone family 3B |  |  | 0.36 |
| NM_003642 | HAT1 | Histone acetyltransferase 1 | -0.4 | -1.12 | -0.36 |
| NM_018486 | HDAC8 | Histone deacetylase 8 | -0.69 |  | -0.55 |
| NM_178423 | HDAC9 | Histone deacetylase |  | -0.72 |  |
| NM_005321 | HIST1H1E | Histone cluster 1 H1e | -1.21 | -1.4 | -0.84 |
| NM_170745 | HIST1H2AA | Histone cluster 1 h2aa |  | -0.71 |  |
| NM_003513 | HIST1H2AB | Histone cluster 1 h2ab | -0.35 |  |  |
| NM_021065 | HIST1H2AD | Histone cluster 1 h2ad |  | 0.57 |  |
| NM_021052 | HIST1H2AE | Histone cluster 1 h2ae | -0.71 |  |  |
| NM_021064 | HIST1H2AG | Histone cluster 1 h2ag | -0.4 |  |  |
| NM_080596 | HIST1H2AH | Histone cluster 1 h2ah | -0.44 |  |  |
| NM_021066 | HIST1H2AJ | Histone cluster 1 h2aj | -0.82 | -0.92 |  |
| NM_003510 | HIST1H2AK | Histone cluster 1 h2ak |  |  |  |
| NM_003514 | HIST1H2AM | Histone cluster 1 h2am | -0.47 |  | -0.5 |
| NM_021062 | HIST1H2BB | Histone cluster 1 h2bb | 0.77 |  |  |
| BQ683489 | HIST1H2BD | Histone cluster 1 h2bd | -1.29 | -1.98 | -0.59 |
| NM_003523 | HIST1H2BE | Histone cluster 1 h2be |  |  |  |
| NM_003522 | HIST1H2BF | Histone cluster 1 h2bf | 0.66 |  |  |
| NM_003524 | HIST1H2BH | Histone cluster 1 h2bh | 0.69 |  |  |
| NM_003525 | HIST1H2BI | Histone cluster 1 h2bi | 0.72 |  | 0.53 |
| BC014312 | HIST1H2BJ | Histone cluster 1 h2bj |  |  | 1.04 |
| NM_003519 | HIST1H2BL | Histone cluster 1 h2bl | 0.65 |  |  |
| NM_003521 | HIST1H2BM | Histone cluster 1 h2bm | 0.67 | 0.63 | 0.42 |
| NM_003527 | HIST1H2BO | Histone cluster 1 h2bo | 0.7 | 0.61 |  |
| NM_003529 | HIST1H3A | Histone cluster 1 H3a | -0.6 |  |  |
| NM_003537 | HIST1H3B | Histone cluster 1 H3b | -1.19 | -1.56 | -0.97 |
| NM_003530 | HIST1H3D | Histone cluster 1 h3d | -0.5 | -0.67 |  |
| NM_021018 | HIST1H3F | Histone cluster 1 H3f | -0.56 | -0.85 |  |
| NM_003534 | HIST1H3G | Histone cluster 1 H3g | -0.67 | -1.61 | -0.48 |
| NM_003536 | HIST1H3H | Histone cluster 1 H3h | -0.49 |  |  |
| NM_003535 | HIST1H3J | Histone cluster 1 H3j | -0.81 |  |  |
| NM_003544 | HIST1H4B | Histone cluster 1 H4b |  | -0.72 |  |
| NM_003543 | HIST1H4H | Histone cluster 1 H4h |  | -0.61 |  |
| NM_003495 | HIST1H4I | Histone cluster 1 H4i |  | -0.87 |  |
| NM_021968 | HIST1H4J | Histone cluster 1 H4j | -0.57 | -1.03 |  |
| NM_001040874 | HIST2H2AA4 | Histone cluster 2 h2aa4 | 0.78 | 0.79 | 0.62 |
| NM_175065 | HIST2H2AB | Histone cluster 2 h2ab | -0.37 | -1.02 | -0.45 |
| NM_003517 | HIST2H2AC | Histone cluster 2 h2ac | -1.62 | -1.68 | -0.94 |
| NM_003528 | HIST2H2BE | Histone cluster 2 h2be | 0.35 |  |  |
| NM_001005464 | HIST2H3A | Histone cluster 2 h3a | -0.91 |  | -0.52 |
| NM_001025303 | HIST2H3PS2 | Histone cluster 2 H3 pseudogene 2 | -0.95 | -1.78 | -0.59 |
| NM_001034077 | HIST2H4B | Histone cluster 2 H4b |  | -0.73 |  |
| DB010344 | HIST3H2BB | Histone cluster 3 h2bb | -1.57 | -2.16 | -0.75 |
| NM_003493 | HIST3H3 | Histone cluster 3 H3 | -0.35 |  |  |
| NM_172164 | NASP | Nuclear autoantigenic sperm protein | -0.46 | -0.64 | -0.44 |
| NM_006315 | PCGF3 | Polycomb group ring finger 3 |  | 0.75 |  |
| NM_032373 | PCGF5 | Polycomb group ring finger 5 | -0.58 |  |  |
| NM_198319 | PRMT1 | Protein arginine methyltransferase 1 | -0.58 | -0.52 | -0.46 |
| NM_206962 | PRMT2 | Protein arginine methyltransferase | 0.53 |  |  |
| NM_015355 | SUZ12 | Suppressor of zeste 12 homolog | -0.7 | -0.94 | -0.65 |
| NM_182931 | MLL5 | Myeloid/lymphoid or mixed-lineage leukemia 5 | -0.84 |  | -0.77 |
|  |  |  |  |  |  |
| Structural |  |  |  |  |  |
| NM_004411 | DYNC1I1 | Dynein cytoplasmic 1 intermediate chain 1 | 0.46 | 0.84 | 0.53 |
| NM_019063 | EML4 | Echinoderm microtubule associated protein like 4 | -1.54 | -0.8 | -0.81 |
| NM_080386 | H2-ALPHA | Alpha-tubulin isotype H2-alpha | -0.86 |  | -0.8 |
| NM_002374 | MAP2 | Microtubule-associated protein 2 transcript variant 1 | 0.51 |  |  |
| NM_003980 | MAP7 | Microtubule-associated protein 7 | -0.6 |  | -0.41 |
| NM_015471 | NSL1 | NSL1 kinetochore complex component 1 |  |  |  |
| NM_145697 | NUF2 | NUF2 NDC80 kinetochore complex component |  | -0.84 |  |
| NM_016359 | NUSAP1 | Nucleolar and spindle associated protein 1 | -0.53 |  | -0.71 |
| NM_006306 | SMC1A | Structural maintenance of chromosomes 1A | -0.66 | -0.66 | -0.6 |
| NM_079836 | TUBA2 | Tubulin alpha 2 transcript variant 2 | -1.11 | -0.56 | -1.11 |
| NM_006009 | TUBA3 | Tubulin alpha 3 | -1.15 |  | -1.02 |
| NM_032704 | TUBA6 | Tubulin alpha 6 | -1.34 |  |  |
| NM_018943 | TUBA8 | Tubulin alpha 8 | -0.62 |  | -0.68 |
| NM_178014 | TUBB | Tubulin beta | -1.07 |  | -0.92 |
| NM_001069 | TUBB2A | Tubulin beta 2A | -0.47 |  | -0.68 |
| NM_006088 | TUBB2C | Tubulin beta 2C | -1.32 | -0.8 | -1.09 |
| NM_006086 | TUBB3 | Tubulin beta 3 | -1.27 | -0.66 | -1.25 |
| NM_006087 | TUBB4 | Tubulin beta 4 | -0.8 |  | -0.53 |
| NM_032525 | TUBB6 | Tubulin beta 6 | -0.67 |  | -0.59 |
| NM_177987 | TUBB8 | Tubulin beta 8 | -1.03 |  | -0.69 |
| NM_001070 | TUBG1 | Tubulin gamma 1 | -0.58 |  | -0.44 |
|  |  |  |  |  |  |
| Regulation of Transcription | |  |  |  |  |
| NM_005194 | CEBPB | CCAAT/enhancer binding protein beta |  | 1.3 |  |
| NM_006079 | CITED2 | Cbp/p300-interacting transactivator with Glu/Asp-rich carboxy-terminal domain 2 | 0.85 | 0.84 |  |
| NM_133467 | CITED4 | Cbp/p300-interacting transactivator with Glu/Asp-rich carboxy-terminal domain 4 |  |  |  |
| NM_182898 | CREB5 | Camp responsive element binding protein 5 transcript variant 1 | 0.47 | 0.71 | 0.52 |
| NM_017779 | DEPDC1 | DEP domain containing 1 |  |  |  |
| NM_004405 | DLX2 | Distal-less homeobox 2 | -0.64 | -1.03 | -0.9 |
| NM_005225 | E2F1 | E2F transcription factor 1 |  |  |  |
| NM_024680 | E2F8 | E2F transcription factor 8 | -0.75 |  |  |
| NM_001964 | EGR1 | Early growth response 1 |  | 1.21 |  |
| NM_005252 | FOS | V-fos FBJ murine osteosarcoma viral oncogene homolog |  | 1.23 |  |
| NM_181054 | HIF1A | Hypoxia-inducible factor 1 alpha subunit transcript variant 2 |  | 0.86 | 0.42 |
| NM_145904 | HMGA1 | High mobility group AT-hook 1 transcript variant 6 | -0.58 |  |  |
| NM_003483 | HMGA2 | High mobility group AT-hook 2 | -0.39 | -0.53 |  |
| NM_002128 | HMGB1 | High-mobility group box 1 | -0.67 | -0.7 | -0.75 |
| NM_002129 | HMGB2 | High-mobility group box 2 |  |  |  |
| NM_000600 | IL6 | Interleukin 6 | 1.68 | 3.66 | 1.93 |
| NM_198219 | ING1 | Inhibitor of growth family member 1 | -0.47 | -0.61 |  |
| NM_002192 | INHBA | Inhibin beta A |  | 1.47 |  |
| NM_004973 | JARID2 | Jumonji AT rich interactive domain 2 | 0.63 | 0.82 | 0.65 |
| NM_002228 | JUN | Jun oncogene |  |  |  |
| NM_016269 | LEF1 | Lymphoid enhancer-binding factor 1 | 0.66 | 0.93 | 0.54 |
| NM_006879 | MDM2 | Mdm2 transformed 3T3 cell double minute | 1.23 | 1.85 | 1.13 |
| NM_031300 | MXD3 | MAX dimerization protein 3 |  | -0.71 | -0.39 |
| NM_006454 | MXD4 | MAX dimerization protein 4 |  |  |  |
| X66087 | MYBL1 | v-Myb myeloblastosis viral oncogene homolog (avian)-like 1 | -0.7 | -1.03 | -0.94 |
| NM_005595 | NFIA | Nuclear factor I/A | -0.75 | -0.72 | -0.96 |
| NM_005450 | NOG | Noggin |  | -0.58 |  |
| NM_021005 | NR2F2 | Nuclear receptor subfamily 2 group F member 2 | -0.91 | -1.33 | -0.77 |
| NM_006191 | PA2G4 | Proliferation-associated 2G4 38kda | -0.53 | -0.6 | -0.54 |
| NM_002691 | POLD1 | Polymerase delta 1 catalytic subunit 125kda |  | -0.55 | -0.62 |
| NM_021144 | PSIP1 | PC4 and SFRS1 interacting protein 1 transcript variant 1 | -0.69 | -0.98 | -0.53 |
| NM_006325 | RAN | RAN member RAS oncogene family | -0.76 |  |  |
| NM_001012761 | RGMB | RGM domain family member B transcript variant 1 | 0.78 | 0.9 | 0.65 |
| NM_012249 | RHOQ | Ras homolog gene family member Q | 0.57 | 0.91 | 0.47 |
| NM_017420 | SIX4 | Sine oculis homeobox homolog 4 | -0.34 | -0.56 | -0.36 |
| NM_022739 | SMURF2 | SMAD specific E3 ubiquitin protein ligase 2 | 0.49 |  |  |
| NM_003107 | SOX4 | SRY -box 4 | 0.55 | 1.92 | 0.42 |
| U46752 | SQSTM1 | Human phosphotyrosine independent ligand | 0.98 | 0.52 |  |
| NM_001005291 | SREBF1 | Sterol regulatory element binding transcription factor | -0.35 |  | -0.64 |
| NM_003168 | SUPT4H1 | Suppressor of Ty 4 homolog 1 | 0.81 | 0.94 | 0.83 |
| NM_139215 | TAF15 | TAF15 RNA polymerase II | -0.63 | -1.41 |  |
| NM_003221 | TFAP2B | Transcription factor AP-2 beta |  |  | 0.47 |
| NM_194259 | UBE2I | Ubiquitin-conjugating enzyme E2I transcript variant 2 |  | -0.53 |  |
| NM_013282 | UHRF1 | Ubiquitin-like containing PHD and RING finger domains | -0.99 | -1.59 | -1.06 |
| NM_003400 | XPO1 | Exportin 1 | -0.58 |  |  |
|  |  |  |  |  |  |
| Translation |  |  |  |  |  |
| NM_001280 | CIRBP | Cold inducible RNA binding protein |  | 0.61 |  |
| NM_001950 | E2F4 | E2F transcription factor 4 p107/p130-binding | -0.45 |  |  |
| NM_032378 | EEF1D | Eukaryotic translation elongation factor 1 delta transcript variant 1 | -0.34 |  |  |
| NM_014239 | EIF2B2 | Eukaryotic translation initiation factor 2B subunit 2 beta 39kda |  |  | -0.41 |
| NM_003907 | EIF2B5 | Eukaryotic translation initiation factor 2B subunit 5 epsilon 82kda |  |  | -0.45 |
| NM_004094 | EIF2S1 | Eukaryotic translation initiation factor 2 subunit 1 alpha 35kda |  | 0.67 |  |
| NM_003755 | EIF3S4 | Eukaryotic translation initiation factor 3 subunit 4 delta 44kda | -0.34 |  |  |
| NM_001568 | EIF3S6 | Eukaryotic translation initiation factor 3 subunit 6 48kda |  | -0.84 |  |
| NM_003753 | EIF3S7 | Eukaryotic translation initiation factor 3 subunit 7 zeta 66/67kda | -0.39 |  | -0.41 |
| NM_001037808 | EIF3S8 | Eukaryotic translation initiation factor 3 subunit 8 110kda |  |  | -0.45 |
| NM_001416 | EIF4A1 | Eukaryotic translation initiation factor 4A isoform 1 | -0.48 |  | -0.55 |
| NM_001967 | EIF4A2 | Eukaryotic translation initiation factor 4A isoform 2 |  | 0.67 |  |
| NM_001417 | EIF4B | Eukaryotic translation initiation factor 4B | -0.42 | 0.71 |  |
| NM_001968 | EIF4E | Eukaryotic translation initiation factor 4E | -0.38 | -1.01 |  |
| NM_182917 | EIF4G1 | Eukaryotic translation initiation factor 4 gamma 1 |  |  | -0.38 |
| NM_006532 | ELL | Elongation factor RNA polymerase II | 0.5 |  |  |
| NM_012081 | ELL2 | RNA polymerase II elongation factor ELL2. | 0.66 | 0.63 |  |
| NM_017572 | MKNK2 | MAP kinase interacting serine/threonine kinase 2 | 0.79 |  |  |
| NM_006191 | PA2G4 | Proliferation-associated 2G4 38kda | -0.53 | -0.6 | -0.54 |
| NM_002568 | PABPC1 | Polybinding protein cytoplasmic 1 | -0.63 |  | -0.51 |
| NM_002691 | POLD1 | Polymerase delta 1 catalytic subunit 125kda |  | -0.55 |  |
| NM_017443 | POLE3 | Polymerase epsilon 3 |  |  | -0.41 |
| NM_003246 | THBS1 | Thrombospondin 1 | 0.47 | 1.14 | 0.5 |
| NM_001033930 | UBA52 | Ubiquitin A-52 residue ribosomal protein fusion product 1 | 0.34 |  |  |
|  |  |  |  |  |  |
| Ras-related |  |  |  |  |  |
| NM_015576 | ERC2 | ELKS/RAB6-interacting/CAST family member 2 |  |  | 0.86 |
| BC034222 | HRASLS5 | HRAS-like suppressor family member 5 | 0.56 |  |  |
| NM_012219 | MRAS | Muscle RAS oncogene homolog | -0.73 |  | -0.78 |
| NM_016131 | RAB10 | RAB10 member RAS oncogene family |  |  | -0.44 |
| NM_014904 | RAB11FIP2 | RAB11 family interacting protein 2 | -0.52 | -0.91 | -0.68 |
| NM_001025300 | RAB12 | RAB12 member RAS oncogene family |  | 0.83 |  |
| NM_016322 | RAB14 | RAB14 member RAS oncogene family |  |  | -0.36 |
| NM_198686 | RAB15 | RAB15 member RAS onocogene family | -0.46 |  |  |
| NM_014999 | RAB21 | RAB21 member RAS oncogene family |  | 0.85 |  |
| NM_001031677 | RAB24 | RAB24 member RAS oncogene family |  |  | 0.36 |
| NM_004794 | RAB33A | RAB33A member RAS oncogene family | -0.72 | -0.95 |  |
| NM_004162 | RAB5A | RAB5A member RAS oncogene family |  | 0.59 |  |
| NM_005370 | RAB8A | RAB8A member RAS oncogene family | -0.52 | -0.68 | -0.48 |
| NM_004703 | RABEP1 | Rabaptin RAB gtpase binding effector protein 1 | 1.59 |  |  |
| NM_014857 | RABGAP1L | RAB gtpase activating protein 1-like |  | 0.56 |  |
| NM_014504 | RABGEF1 | RAB guanine nucleotide exchange factor | 0.5 | 0.68 |  |
| NM_004582 | RABGGTB | Rab geranylgeranyltransferase beta subunit |  |  | 0.42 |
| NM_002871 | RABIF | RAB interacting factor | 0.57 |  |  |
| NM_173825 | RABL3 | RAB member of RAS oncogene family-like 3 |  | -0.52 |  |
| NM_006860 | RABL4 | RAB member of RAS oncogene family-like 4 |  | -0.62 |  |
| NM_013277 | RACGAP1 | Rac GTPase activating protein 1 |  |  |  |
| NM_006325 | RAN | RAN member RAS oncogene family |  | -0.76 |  |
| NM_006989 | RASA4 | RAS p21 protein activator 4 |  | 0.62 |  |
| NM_032023 | RASSF4 | Ras association domain family 4 |  |  | -0.57 |
| NM_032023 | RASSF4 | Ras association domain family 4 |  |  |  |
| NM_007211 | RASSF8 | Ras association domain family 8 | 0.65 |  |  |
| NM_000539 | RHO | Rhodopsin |  |  | 0.6 |
| NM_004040 | RHOB | Ras homolog gene family member B | 0.45 |  |  |
| NM_175744 | RHOC | Ras homolog gene family member C |  | 0.77 |  |
| NM_012249 | RHOQ | Ras homolog gene family member Q | 0.72 | 0.91 | 0.39 |
| NM_005168 | RND3 | Rho family gtpase 3 |  | 0.85 | -0.63 |
| NM_004165 | RRAD | Ras-related associated with diabetes | 1.38 | 0.56 | 0.69 |
| NM_006270 | RRAS | Related RAS viral | 0.53 |  |  |
| NM_012250 | RRAS2 | Related RAS viral oncogene homolog 2 | 0.48 | 1.15 |  |
|  |  |  |  |  |  |
| Extracellular matrix | |  |  |  |  |
| NM_001110 | ADAM10 | ADAM metallopeptidase domain 10 |  | 0.48 |  |
| NM_001005845 | ADAM9 | ADAM metallopeptidase domain 9 transcript variant 2 | 0.56 | 1.21 |  |
| NM_014243 | ADAMTS3 | ADAM metallopeptidase with thrombospondin type 1 motif 3 | 0.39 | 0.85 |  |
| NM_014272 | ADAMTS7 | ADAM metallopeptidase with thrombospondin type 1 motif 7 | -0.52 | 0.67 |  |
| NM_014272 | ADAMTS7 | ADAM metallopeptidase with thrombospondin type 1 motif 7 |  |  |  |
| NM_001792 | CDH2 | Cadherin 2 type 1 N-cadherin |  | 0.7 |  |
| NM_022478 | CDH24 | Cadherin-like 24 transcript variant 1 | -0.91 |  |  |
| NM_006383 | CIB2 | Calcium and integrin binding family member 2 | 0.55 |  | 0.39 |
| NM_054113 | CIB3 | Calcium and integrin binding family member 3 | 0.41 | 0.5 |  |
| NM_001903 | CTNNA1 | Catenin alpha 1 102kda |  | 0.55 |  |
| NM_001904 | CTNNB1 | Catenin beta 1 88kda |  | 0.68 |  |
| ENST00000320216 | ENST00000320216 | Integrin beta-2 precursor |  |  |  |
| NM_003862 | FGF18 | Fibroblast growth factor 18 | 0.94 |  |  |
| NM_212482 | FN1 | Fibronectin 1 transcript variant 1 | 0.61 |  |  |
| NM_005114 | HS3ST1 | Heparan sulfate 3-O-sulfotransferase 1 | -0.72 | 1.04 | 0.87 |
| NM_006043 | HS3ST2 | Heparan sulfate 3-O-sulfotransferase 2 | 0.38 |  |  |
| NM_006040 | HS3ST4 | Heparan sulfate 3-O-sulfotransferase 4 |  | 0.99 |  |
| NM_001077188 | HS6ST2 | Heparan sulfate 6-O-sulfotransferase 2 transcript variant L |  | 0.62 |  |
| NM_030790 | ITFG1 | Integrin alpha FG-GAP repeat containing 1 | 0.43 |  |  |
| NM_002205 | ITGA5 | Integrin alpha 5 |  |  | -0.4 |
| NM_002210 | ITGAV | Integrin alpha V |  |  | -1.83 |
| NM_133376 | ITGB1 | Integrin beta 1 transcript variant 1E | 0.41 | 0.71 |  |
| NM_014288 | ITGB3BP | Integrin beta 3 binding protein |  | -0.76 |  |
| NM_002213 | ITGB5 | Integrin beta 5 |  | -0.65 |  |
| NM_002214 | ITGB8 | Integrin beta 8 |  |  | -0.5 |
| NM_002421 | MMP1 | Matrix metallopeptidase 1 | 1.93 |  |  |
| NM_002427 | MMP13 | Matrix metallopeptidase 13 |  | 0.57 | 0.73 |
| NM_016155 | MMP17 | Matrix metallopeptidase 17 | -0.41 | 0.64 |  |
| NM_002429 | MMP19 | Matrix metallopeptidase 19 |  | 0.82 |  |
| NM_004994 | MMP9 | Matrix metallopeptidase 9 |  | 0.53 |  |
| NM_004994 | MMP9 | Matrix metallopeptidase 9 |  | 0.55 |  |
| NM_005947 | MT1B | Metallothionein 1B | 0.77 | 0.66 | 0.38 |
| NM_175617 | MT1E | Metallothionein 1E | 0.78 | 0.68 |  |
| NM_005950 | MT1G | Metallothionein 1G | 0.6 |  |  |
| NM_005951 | MT1H | Metallothionein 1H | 0.78 | 0.59 |  |
| NM_005952 | MT1X | Metallothionein 1X | 0.81 | 0.6 |  |
| NM_198883 | MTX1 | Metaxin 1 | -0.33 |  |  |
| NM_003255 | TIMP2 | TIMP metallopeptidase inhibitor 2 | 1.38 | 1.18 | 0.4 |
|  |  |  |  |  |  |
| Protein folding |  |  |  |  |  |
| AY956764 | HSP90AB3P | Heat shock protein 90Bc | -1.14 |  | -0.59 |
| NM_006819 | STIP1/HOP | Stress-induced-phosphoprotein 1 | -0.58 |  | -0.47 |
| ENST00000378770 | ENST00000378770 | Heat shock protein 90Ad. |  | -0.86 |  |
| NM_001040141 | HSP90AA2 | Heat shock protein 90kda alpha , class A member 2 | -0.65 | -0.74 | -0.69 |
| NM_001540 | HSPB1 | Heat shock 27kda protein 1 | 0.49 |  |  |
| NM_002155 | HSPA6 | Heat shock 70kda protein 6 | 1.5 |  |  |
| NM_003299 | HSP90B1 | Heat shock protein 90kda beta member 1 | -1.54 |  | -1.34 |
| NM_005347 | HSPA5 | Heat shock 70kDa protein 5 |  | 1.07 |  |
| NM_005348 | HSP90AA1 | Heat shock protein 90kDa alpha (, class A member 1 |  |  | -0.92 |
| NM_007034 | DNAJB4 | DnaJ homolog, subfamily B, member B4 | 0.51 |  |  |
| NM_007355 | HSP90AB1 | heat shock protein 90kDa alpha class B member 1 | -1.35 |  | -0.8 |
| NM_013238 | DNAJC15 | DnaJ homolog, subfamily C, member 15 | 0.43 |  |  |
| NM_014280 | DNAJC8 | DnaJ homolog, subfamily C, member 8 | -0.48 |  |  |
| NM_014787 | DNAJC6 | DnaJ homolog, subfamily C, member 6 | 0.64 | 0.63 |  |
| NM_015268 | DNAJC13 | DnaJ homolog, subfamily C, member 13 |  |  | -0.43 |
| NM_017626 | DNAJB12 | DnaJ homolog, subfamily B, member 12 |  | -0.67 |  |
| NM_018981 | DNAJC10 | DnaJ homolog, subfamily C, member 10 |  |  | -0.47 |
| NM_021800 | DNAJC12 | DnaJ homolog, subfamily C, member 12 |  |  | -0.41 |
| NM_021979 | HSPA2 | Heat shock 70kDa protein 2 | -0.43 |  | -0.67 |
| NM_152686 | DNAJC18 | DnaJ homolog, subfamily C, member 18 | 0.49 | 0.56 |  |
| NM_173650 | DNAJC5G | DnaJ homolog, subfamily C, member 5G | 0.45 |  |  |
|  |  |  |  |  |  |
