# Supplementary material for: Signaling Pathways of ESE-16, an Antimitotic and Anticarbonic Anhydrase Estradiol Analog, in Breast Cancer Cells
Source: PLoS One. 2013 Jan 31;8(1):e53853. doi: 10.1371/journal.pone.0053853 (PMC3561402; doi:10.1371/journal.pone.0053853)
Supplement: Supporting Information S3 — Bcl-2 flow cytometry data. Percentage of cells in the FI unit ranges of 0–7.5, 7.51–75 and 75.1–1000 as an indication of the quantity of Bcl-2 (Ser 70) phosphorylation per cell. (DOCX) [file pone.0053853.s003.docx]

| **Cell line** | **Description** | **FI units (7.51-75)** | | **FI units (0-7.5)** | | **FI units (75.1-1000)** | |
| --- | --- | --- | --- | --- | --- | --- | --- |
|  |  | **Average** | **STDEV** | **Average** | **STDEV** | **Average** | **STDEV** |
| **MCF-7** | **Vehicle-treated** | 91.26 | 2.9 | 2.51 | 0.9 | 6.27 | 2.1 |
|  | **ESE-16-treated control** | 19.67 | 3.4 | 46.85 | 3.7 | 33.43 | 2.3 |
|  | **ESE-16 + SP600125 (JNKi)-treated** | 58.59 | 7.3 | 21.79 | 5.3 | 19.64 | 4.0 |
|  | **ESE-16 + SB239063 (p38i)-treated** | 30.13 | 4.3 | 29.50 | 6.1 | 39.70 | 2.9 |
|  |  |  |  |  |  |  |  |
| **MDA-MB-231** | **Vehicle-treated** | 96.26 | 0.5 | 2.59 | 0.2 | 1.14 | 0.3 |
|  | **ESE-16-treated control** | 23.69 | 1.8 | 51.42 | 3.0 | 24.87 | 1.5 |
|  | **ESE-16 + SP600125 (JNKi)-treated** | 78.76 | 3.9 | 16.00 | 5.1 | 5.24 | 2.6 |
|  | **ESE-16 + SB239063 (p38i)-treated** | 36.26 | 2.1 | 37.87 | 2.0 | 25.87 | 0.9 |
|  |  |  |  |  |  |  |  |
|  | **Vehicle-treated** | 94.54 | 1.3 | 1.26 | 0.7 | 4.20 | 1.6 |
| **MCF-12A** | **ESE-16-treated control** | 45.10 | 3.4 | 24.95 | 7.5 | 29.94 | 4.6 |
|  | **ESE-16 + SP600125 (JNKi)-treated** | 52.65 | 8.8 | 27.64 | 8.3 | 19.61 | 0.8 |
|  | **ESE-16 + SB239063 (p38i)-treated** | 39.05 | 1.4 | 15.49 | 0.9 | 45.44 | 1.6 |
|  |  |  |  |  |  |  |  |
